# Improved survival in patients with refractory variceal bleeding treated with esophageal stents: A multicenter cohort study

**DOI:** 10.1016/j.jhepr.2025.101581

**Published:** 2025-08-30

**Authors:** Delphine Weil, Morgane Clément, Charlotte Bouzbib, Jean-Paul Cervoni, Andrimalala Raoto, Grégoire Boivineau, Isabelle Ollivier-Hourmand, Noémi Reboux, Caroline Lemaitre, Cassandra Rayer, Marine Camus-Duboc, Ludovic Caillo, André-Jean Remy, Laure Elkrief, Guillaume Conroy, Faustine Wartel, Armand Garioud, Maeva Guillaume, Edouard Bardou-Jacquet, Stéphane Koch, Jean-Pierre Arpurt, Marika Rudler, Vincent Di Martino, ANGH, CREGG, SFED, GRAPHE, CFHTP groups

**Affiliations:** 1 Service d’Hépatologie et Soins Intensifs Digestifs, CHU Jean Minjoz, Besançon, France; 2 Université Marie et Louis Pasteur, EFS, INSERM UMR1098 RIGHT, Besançon, F-25000, France; 3 Unité de Soins Intensifs d'Hépatologie et Gastro-Entérologie, Hôpital Pitié-Salpêtrière, APHP, Paris, France; 4 Service d’Hépato-Gastroentérologie, CH d’Avignon, Avignon, France; 5 Service de Gastroentérologie, Hôpital Nord, AP-HM, Hôpital Nord AP-HM, Marseille, France; 6 Service d’Hépatologie, CHU Côte de Nacre, Caen, France; 7 Service d’Hépato-Gastroentérologie, CHU Cavale Blanche, Brest, France; 8 Service d’Hépato-Gastroentérologie, Groupe Hospitalier du Havre, Le Havre, France; 9 Service des Maladies du Foie, CHU de Rennes, France; 10 Centre d'Endoscopie Digestive, DMU SAPERE, Sorbonne Université, Hôpital Saint-Antoine AP-HP, Paris, France; 11 Service d'Hépato-Gastroentérologie, CHU de Nîmes, Université de Montpellier-Nimes, Nîmes, France; 12 Service d’Hépato-Gastroentérologie et Maladies de la Nutrition, CH de Perpignan, Perpignan, France; 13 Service d'Hépato-Gastroentérologie, Hôpital Trousseau - CHU de Tours, Tours, France; 14 Service d'Hépato-Gastroentérologie, CHR de Metz-Thionville Hôpital Mercy, Metz, France; 15 Service des Maladies de l’Appareil digestif et de la Nutrition, CH de Valenciennes, Valenciennes, France; 16 Service d'Hépato-Gastroentérologie, CHI Lucie et Raymond Aubrac, Villeneuve-Saint-Georges, France; 17 Service de Gastro-Entérologie, Clinique Pasteur, Toulouse, France; 18 Université de Rennes, INSERM Institut Numecan, Rennes, France; 19 Service de Gastroentérologie et Nutrition, Endoscopie Digestive, CHU Jean Minjoz, Besançon, France

**Keywords:** Portal hypertension, Esophageal varices, Tamponade devices, Rescue transjugular intrahepatic portosystemic shunt (rTIPS), Mortality

## Abstract

**Background & Aims:**

Tamponade is a bridge therapy for refractory variceal bleeding. This study compared esophageal stents (ESs) and balloon tamponade (BT) in terms of early bleeding control and mortality.

**Methods:**

We analyzed a cohort of patients with cirrhosis treated with tamponade in nine French hospitals between 2002 and 2023. The primary outcome was 6-week mortality. Multivariable analyses included Cox with time-dependent covariates and logistic regression models, adjusted for model for end-stage liver disease (MELD) score, rescue transjugular intrahepatic portosystemic shunt (rTIPS), and inverse probability of treatment weighting (IPTW) for ES.

**Results:**

Sixty-three patients (87.3% male; mean age 55 years; 73.0% Child-Pugh C) were included. ES was used in 30 patients, BT in 33, and rTIPS subsequently in 20. Endoscopic control was attempted in 66.1% of cases. Adverse events were more frequent with ES (56.7% *vs.* 27.3%, *p* = 0.018), mostly stent migrations without clinical consequence, but less severe than with BT (two esophageal ruptures). Mortality was 42.9% (n = 27) at Day 5 and 55.6% (n = 35) at 6 weeks. Univariable analysis revealed lower mortality with ES than with BT (20.0% *vs.* 63.6% on Day 5, *p* = 0.0005 and 36.7% *vs.* 72.7% on Week 6, *p* = 0.0015) and with rTIPS (10.0% *vs.* 58.1% on Day 5, *p* = 0.0003 and 25.5% *vs.* 67.5% on Week 6, *p* = 0.0009). IPTW-adjusted and IPTW-weighted time-dependent Cox and logistic models confirmed decreased 6-week mortality (hazard ratio (HR) = 0.26, *p* = 0.0078) and better 5-day bleeding control (odds ratio (OR) = 6.1, *p* = 0.020) with ES.

**Conclusions:**

In patients with cirrhosis-related refractory variceal bleeding, ESs were associated with significantly better early bleeding control and reduced mortality at both Day 5 and Week 6 compared with BT. Prospective studies are warranted to confirm these findings.

**Impact and implications:**

Tamponade devices are crucial in the management of refractory variceal bleeding, with balloon probes being the only option until the early 2000s. More recently, self-expanding ESs have been recommended, although their superiority over BT in terms of survival has not yet been demonstrated. Our multicenter cohort study, using propensity score analysis, found that ESs provided superior bleeding control at Day 5 and survival benefit at Week 6, regardless of severity of liver failure or use of rescue TIPS. These findings highlight ESs as the optimal tamponade device, although they might not obviate the need for prompt rescue TIPS placement.

## Highlights

• After tamponade, all-cause mortality was 42.9% at Day 5 and 55.6% at week six.
• Both ES (rather than BT) and rTIPS improved bleeding control at day five.
• Six-week mortality was lower with ES *vs.* BT and in patients who underwent rTIPS.
• ES plus rTIPS provided the highest survival rate (91.7% at day five; 75.0% at week six).

## Graphical abstract

**Figure:**
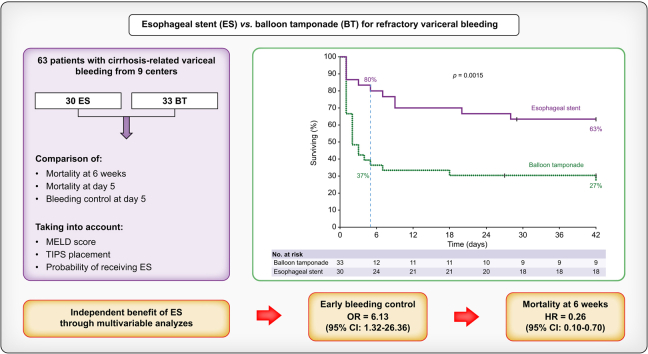

## Introduction

Tamponade devices are used as a temporary measure in the event of refractory variceal bleeding, either massive at the outset or recurrent within the first 5 days, pending the availability of a more efficacious treatment of bleeding and/or portal hypertension.^1^^,^^2^ Currently, two types of device are available: single (Linton) or double (Blakemore) balloon probes[3], [4], [5] and, more recently, expansive esophageal stents (ESs).[6], [7], [8], [9]

In its most recent report, the Baveno Consortium suggested that the use of ESs was the optimal option, although it acknowledged that there were insufficient studies comparing the two categories of tamponade device.^10^ Indeed, the literature reports only one randomized trial that included a very small number of patients, which was unable to demonstrate the superiority of either device in terms of immediate bleeding control or short- or mid-term survival.^11^ The main arguments in favor of the use of ESs over balloons are their ease of placement, longer maintenance period, favorable safety profile, and the absence of esophageal lumen obstruction.^2^^,^^12^

Therefore, we conducted a comparative study to assess whether one device (balloon tamponade [BT] *vs.* ES) is superior to another in terms of short- and mid-term survival and bleeding control.

## Patients and methods

### Study design

This was a French multicenter cohort study that considered for inclusion patients from six general hospitals and nine university hospitals. A questionnaire was made available online to all French hepato-gastroenterologists from March 2021 to June 2023 by five French scientific societies involved in portal hypertension and digestive endoscopy, which promoted this study. Inclusions were both retrospective and prospective to ensure a sufficient number of patients and to enable comparison of tamponade devices, covering a period from January 2002 to April 2023.

### Ethics statement

The research was conducted in accordance with the Declarations of Helsinki and Istanbul, and the French Regulatory Authority for clinical studies. The Institutional Board ‘Clinical Research and Innovation Department’ and the Human Protection Committee East Area II Besancon, France, approved this study. A certificate from the President of the Human Protection Committee East Area II Besancon, France indicates that, according to the French Regulatory Authority for Clinical Studies, prospective and retrospective observational studies are not evaluated by Human Protection Committees. No informed consent was required for this study based on French Regulatory Authority guidance.

### Outcome measures

Mortality at Week 6 was the primary endpoint. The secondary endpoints were 5-day mortality, 5-day bleeding control, bleeding recurrence, and tamponade device-related adverse events (AEs). Failure to control the initial bleeding is defined as massive bleeding or early rebleeding within the first 5 days after the index bleeding.^10^ Therefore, we defined recurrent bleeding as occurring after Day 5 and collected these data on Day 7 and Week 6. Device-related AEs were recorded during insertion and after removal. Owing to the variable length of device maintenance, data were collected on Day 7.

### Population

All patients who underwent tamponade for variceal bleeding between March 2021 and April 2023 and met the inclusion criteria were prospectively included in the study. The inclusion criteria were as follows: age >18 years; esophageal variceal bleeding requiring hospitalization and refractory to conventional treatment, and use of tamponade with a balloon catheter or covered self-expanding ES. Patients included in a center reporting the use of a single tamponade device were excluded from the study. Additional exclusion criteria included the existence of gastric varices, non-cirrhotic portal hypertension, or hepatocellular carcinoma. To increase the number of patients, investigators were encouraged to retrospectively include additional consecutive patients who had been hospitalized at their center between 2002 and 2021 based on data from the French National Uniform Hospital Discharge Data Set Database (PMSI) (International Classification of Diseases (ICD)-10 code: EHBD001). However, the timeframe used for the retrospective inclusion was based on the judgment of each investigator. The diagnosis of cirrhosis was based on conventional clinical, biochemical, radiological, or histological criteria. The severity of cirrhosis at the time of bleeding was assessed using the Child-Pugh and model for end-stage liver disease (MELD) scores.

### Data collection

The questionnaire included 177 variables, of which 101 were collected on admission, 49 on Day 7, and 27 at Week six. Three variables were demographic, 18 were related to history of portal hypertension, 53 to characteristics and management of bleeding before tamponade, 23 to tamponade modalities, and 80 to outcome after tamponade. Of the post-tamponade follow-up data, 10 variables were related to AEs associated with the tamponade device, four to bleeding recurrence, and six to mortality. In the event of death, the investigator reported the cause. The use of rescue transjugular intrahepatic portosystemic shunt (rTIPS), defined as TIPS placement within the first 5 days to control bleeding, was also recorded and included four variables.

### Statistical analysis

Results were compared between the BT and ES groups. Quantitative variables were expressed as mean ± SD when normally distributed, and qualitative variables as absolute numbers and percentages. Univariable comparisons were performed using the Student’s *t* test or Mann-Whitney *U* test for quantitative variables, and the Chi-square or Fisher’s exact test for qualitative variables, as appropriate. Survival analyses were conducted using the Kaplan-Meier method, with group comparisons assessed by the Log-rank test.

To adjust for baseline differences and minimize confounding factors, we used multivariable Cox and logistic regression models with inverse probability of treatment weighting (IPTW), derived from a selected propensity score (PS) model (PS3; Materials and methods S1A, S1B, S1C, Table S1, Table S2). The primary analyses were based on a stabilized and truncated IPTW (stIPTW3), applied directly to the models to improve covariate balance while limiting variance inflation. In addition, stIPTW3 was also incorporated as a covariate in separate sensitivity models to explore the consistency of results across alternative adjustment strategies. A time-dependent Cox model was used to account for the immortal time bias introduced by rTIPS placement when analyzing 6-week survival. Variables included in the multivariable models were chosen based on clinical relevance, univariable associations, and the absence of collinearity, adhering to the 10 events per variable rule.

In addition to models using different adjustment strategies (unadjusted, PS-adjusted, and alternative IPTW specifications; Tables S2 and S6), sensitivity analyses were conducted in two contexts. First, the analysis was restricted to patients included during the period when ESs were available (December 2010 to April 2023) to ensure treatment equipoise across centers (Fig. S2 and Table S5A). Second, to address immortal time bias related to delayed transjugular intrahepatic portosystemic shunt (TIPS) placement, a landmark analysis was performed that excluded all patients who died before Day 2 (Fig. S3 and Table S5B). All analyses were performed using NCSS 2019 (Kaysville, UT, USA) and R software, version 4.5.0.

## Results

### Study population

#### Characteristics

**Fig. 1:**
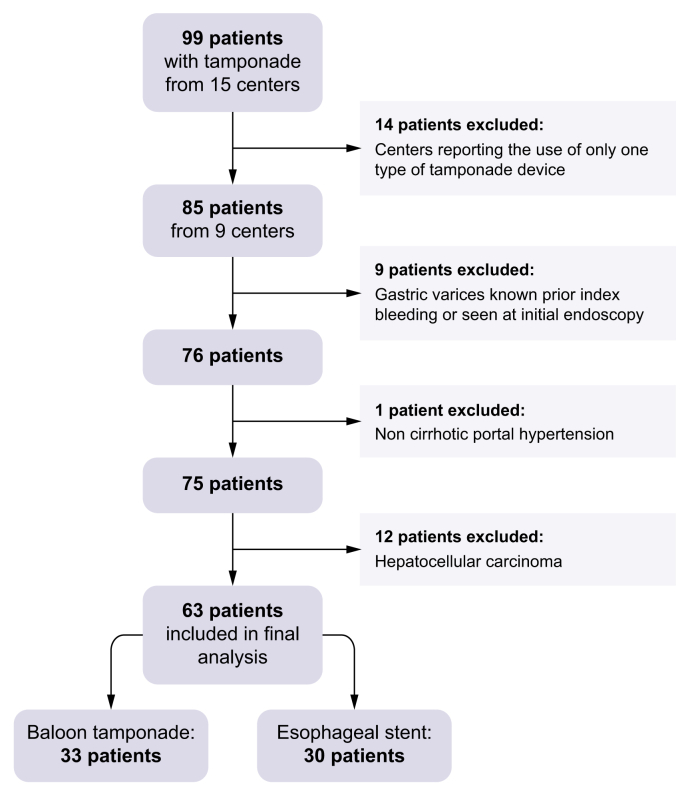
Study flow chart. Flow chart showing the selection process for inclusion in the nationwide multicenter cohort. Among 99 patients with tamponade for variceal bleeding identified across 15 centers, 63 patients fulfilled the eligibility criteria and were included in the analysis. Reasons for exclusion are detailed, including patients from centers reporting the use of only one type of tamponade device, non-cirrhotic portal hypertension, gastric varices, and hepatocellular carcinoma.

The study included 63 patients (33 in the BT group and 30 in the ES group) who were selected from 99 patients recorded from 15 centers (Fig. 1). All of the included patients came from the nine centers that reported using both ES and BT for tamponade (Fig. S1 shows inclusion timeline by center and device availability). The patients had cirrhosis-related portal hypertension and no gastric varices or hepatocellular carcinoma (Fig. 1). The baseline characteristics of the study population are shown in Table 1. Three centers were able to use ES before March, 2021 (in 11 patients). The other centers used ES after they became eligible for reimbursement in 2021. Of the 63 patients, 31 were enrolled prospectively (19 ES *vs.* 12 BT) and 32 retrospectively (11 ES *vs.* 21 BT). The proportion of patients receiving ES was higher among those enrolled prospectively *vs.* those enrolled retrospectively (61.3% *vs.* 38.7%, respectively; *p* = 0.032). Most patients were men (87.3%), with a mean age of 55 ± 13 years. Cirrhosis was alcohol related in 57 patients (90.5%). Most patients (73.0%) had Child-Pugh class C cirrhosis. The mean MELD score was 22.1 ± 9.0. Esophageal varices were documented in 61.9% of patients before the index bleeding episode. Thirty-one patients (49.2%) had previously experienced at least one variceal bleeding event. Of these patients, 15 met the criteria for pre-emptive (p)TIPS^13^ at the time of previous bleeding, and five received TIPS. Of these five patients, three had clinical ascites despite TIPS and one had TIPS thrombosis. The median time between the two bleeding episodes was 13 days for patients with pTIPS criteria during the first episode *vs.* 133 days for other patients. No patient received anticoagulant treatment, whereas only two patients in each group received antiplatelet agents. Baseline hemodynamic parameters were comparable between the two groups. A mean arterial pressure (MAP) <60 mmHg was observed in 31.6% of cases. Mean hemoglobin concentration was 7.1 ± 2.2 g/dl, and mean creatinine was 113 ± 88 μmol/L.

**Table 1:** Baseline characteristics.

Variable	BT (n = 33)	ES (n = 30)	Total (n = 63)	*p* value
Male sex	28 (84.8%)	27 (90.0%)	55 (87.3%)	0.540
Age (years)	55.1 ± 12.5	55.3 ± 12.8	55.2 ± 12.6	0.940
Prospective enrollment	12 (36.4%)	19 (63.3%)	31 (49.2%)	0.032
Alcohol-related cirrhosis	27 (90.9%)	30 (90.0%)	57 (90.5%)	0.902
MELD score	20.6 ± 8.9	23.7 ± 8.9	22.1 ± 9.0	0.172
Child-Pugh A stage	2 (6.1%)	0 (0.0%)	2 (3.2%)	0.270
Child-Pugh B stage	5 (15.1%)	10 (33.3%)	15 (23.8%)	0.091
Child -Pugh C stage	26 (78.8%)	20 (66.7%)	46 (73.0%)	0.279
Known esophageal varices	19 (57.6%)	20 (66.7%)	39 (61.9%)	0.458
Known previous decompensation	19 (57.6%)	24 (80.0%)	43 (68.2%)	0.056
History of variceal bleeding	15 (45.4%)	16 (53.3%)	31 (49.2%)	0.532
Previous TIPS	1 (3.0%)	4 (13.3%)	5 (7.9%)	0.131
Non-selective beta blockers	8 (24.2%)	10 (33.3%)	18 (28.6%)	0.425
Diuretics	7 (21.2%)	10 (33.3%)	17 (27.0%)	0.279
Antiplatelet agents	2 (6.1%)	2 (6.7%)	4 (6.3%)	0.921
Ascites	18/32 (56.2%)	19 (63.3%)	37/62 (56.4%)	0.569
Hepatic encephalopathy	18/32 (56.2%)	17 (56.7%)	35/62 (51.0%)	0.535
Mean arterial pressure (mmHg)	64.1 ± 21.8	65.7 ± 24.6	68.4 ± 18.0	0.790
Hemoglobin (g/dl)	7.7 ± 2.4	6.6 ± 1.9	7.1 ± 2.2	0.051
Prothrombin time (%)	38.9 ± 18.0	33.6 ± 16.3	36.3 ± 17.3	0.222
Bilirubin (μmol/L)	92.6 ± 101.0	121.2 ± 170.1	106.7 ± 138.9	0.425
Albumin (g/L)	25.0 ± 6.8	24.7 ± 4.9	24.8 ± 5.9	0.834
Creatinine (μmol/L)	105.7 ± 73.1	121.2 ± 102.7	113.3 ± 88.5	0.499

Statistical differences were assessed using Chi-squared or Fisher's exact tests for percentages and Student t test for mean differences. BT, balloon tamponade; ES, esophageal stent; MELD, model for end-stage liver disease; TIPS, transjugular portosystemic shunt.

**Fig. 2:**
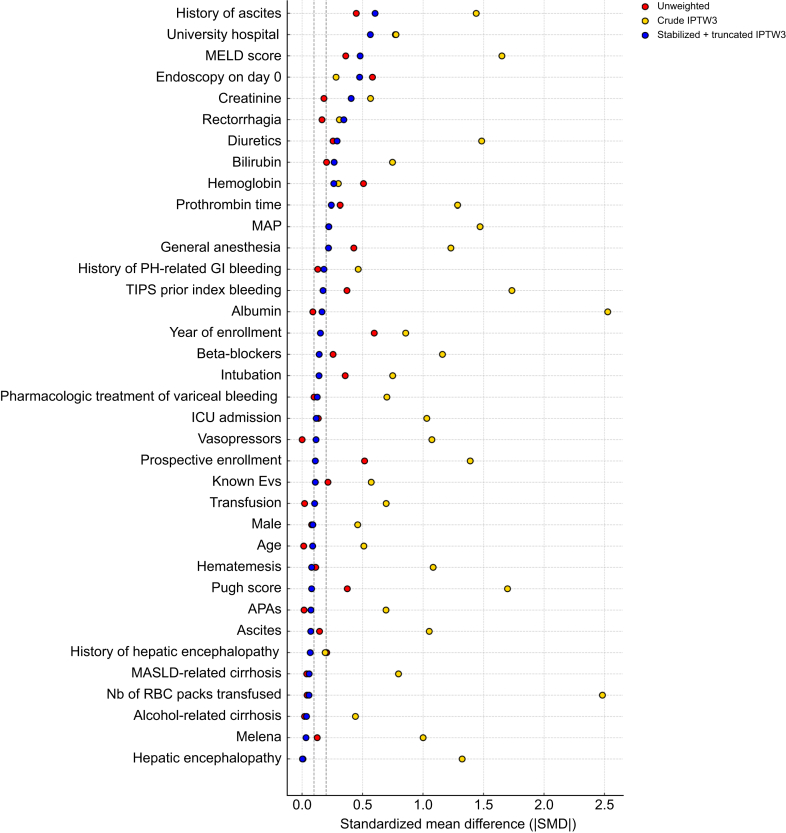
Covariate balance before and after IPTW derived from PS model 3. Love plot showing SMDs for 36 baseline covariates before weighting (red), after crude IPTW using PS3 (yellow), and after stabilized/truncated IPTW using PS3 (blue). SMDs were calculated as mean or proportion differences divided by pooled standard deviation. While crude IPTW failed to correct baseline imbalances, the final weighting strategy substantially improved covariate balance (SMD <0.2 for 23/36 variables; only two variables had SMD >0.5), including key factors, such as year of inclusion and prospective enrollment. The choice of PS3 among 11 PS models is detailed in Table S1. Full balance metrics are provided in Table S2. APA, antiplatelet agent; EV, esophageal varices; GI, gastrointestinal; ICU, intensive care unit; IPTW, inverse probability of treatment weighting; MAP, mean arterial pressure; MASLD, metabolic dysfunction-associated steatotic liver disease; MELD, model for end-stage liver disease; PH, portal hypertension; PS, propensity score; RBC, red blood cell; SMD, standardized mean difference; TIPS, transjugular intrahepatic portosystemic shunt.

The baseline characteristics of patients in both groups served as the basis for constructing multiple PS models to predict the probability of receiving ES *vs.* BT and to select the model that best balanced covariates between groups (Materials and methods S1). The effectiveness of the final weighting strategy, based on stIPTW3, is illustrated in Fig. 2.

#### First-line management of the bleeding episode

Management before tamponade is summarized in Table 2. More than half of the patients (52.4%) were admitted directly to the intensive care unit (ICU). Most patients (90.5%) required blood transfusion, with a mean number of packed red cells of 5.0 units. Antibiotic prophylaxis was prescribed to 57 of 63 patients. On admission, 57 patients underwent endoscopy. This procedure was performed in sufficiently stable patients, and more frequently in the ES group than in the BT group (100.0% *vs.* 81.8%, *p* = 0.014). Endoscopic findings confirmed the presence of grade II (42.5%) or III (55.3%) esophageal varices. Endoscopy performed before tamponade confirmed the esophageal origin of bleeding in all patients. Endoscopic hemostasis was attempted in 70.4% of patients in the BT group compared with 62.1% of patients in the ES group (*p* = 0.512). The most common method of hemostasis was band ligation, used in 32 patients. Post-banding ulcers were diagnosed in 11 patients, with a higher prevalence in the ES group than in the BT group (31.0% *vs.* 7.4%, respectively; *p* = 0.026). The proportion of patients with persistent bleeding despite endoscopic and pharmacological treatment (rather than massive bleeding) did not significantly differ between the ES and BT groups (56.7% *vs.* 45.2%, respectively; *p* = 0.369). The interval between the onset of variceal bleeding and placement of the tamponade device was 14 ± 28 h in the ES group and 12 ± 22 h in the BT group (*p* = 0.661). The time required to place the tamponade device (data available from only 14 patients) was longer in the ES group than in the BT group (16 ± 7 min *vs.* 8 ± 3 min; *p* = 0.021). The technical success rate of tamponade was 93.9% in the BT group and 93.3% in the ES group (*p* = 0.922). Technical difficulties with the self-expanding stent were reported in three cases: one probe was deemed too rigid to pass through the esophageal lumen, one migrated immediately during insertion, and another could not be released because of a lack of gastric balloon expansion. Two instances of failed balloon probe insertion were reported, including one case of esophageal rupture. Overall, two patients in whom the ES procedure failed received a Blakemore probe, and one patient in whom the BT procedure failed received an ES. Given that conversion was performed immediately, assignment to the ES or BT group was based on the most recent device placed. The tamponade device was maintained for an average of 1.5 ± 2.0 days in the BT group, compared with 6.7 ± 4.3 days in the ES group (*p* = 0.001).

**Table 2:** Initial management of bleeding before tamponade.

Variables	BT	ES	Total	*p* value
Care in a university hospital	30 (90.9%)	17 (56.7%)	47 (74.6%)	0.002
Intensive Care Unit admission	16 (48.5%)	17 (56.7%)	33 (52.4%)	0.516
Blood transfusion	30 (90.9%)	27 (90.0%)	57 (90.5 %)	0.903
Number of red blood cell packs (mean ± SD)	5.2 ± 4.5	4.7 ± 3.0	5.0 ± 3.8	0.613
Vasopressive amines	16 (48.5%)	15 (50.0%)	31 (49.2%)	0.904
Vasoactive drugs for portal hypertension	30 (90.9%)	28 (93.3 %)	58 (92.1%)	0.722
Antibiotic prophylaxis	28 (84.8%)	29 (96.7%)	57 (90.5%)	0.111
Intubation	17 (51.5%)	15 (50.0%)	32 (50.8%)	0.904
Endoscopy at day 0	27 (81.8%)	30 (100.0%)	57 (90.5%)	0.014
Esophageal varices stage III∗	15 (55.5%)	16 (55.1%)	31 (55.3%)	0.977
Active bleeding∗	25 (92.6%)	23 (79.3%)	48 (85.7%)	0.156
Adherent clot∗	6 (22.2%)	4 (13.8%)	10 (17.9%)	0.410
Platelet clot∗	3 (11.1%)	7 (24.1%)	10 (17.9%)	0.203
Post banding ulcer∗	2 (7.4%)	9 (31.0%)	11 (19.6%)	0.026
Gastropathy∗	2 (7.4%)	7 (24.1%)	9 (16.1%)	0.088
Endoscopic hemostasis attempt∗	19 (70.4%)	18 (62.1%)	37 (66.1%)	0.512
Band ligation∗	17 (63.0%)	15 (51.7 %)	32 (57.1%)	0.396
Glue obliteration∗	0 (0.0%)	1 (3.4%)	1 (1.8%)	0.834
Sclerosis∗	5 (18.5%)	1 (3.4%)	6 (10.7%)	0.068

Statistical differences were assessed using Chi-squared or Fisher's exact tests for percentages and Student t test for mean differences. BT, balloon tamponade; ES, esophageal stent; ICU, intensive care unit.

∗ Data reported for 56 patients (27 in BT group; 29 in ES group).

#### Rescue TIPS placement

The decision to implant rTIPS was made at the discretion of the referring physician. None of the available baseline variables, including indicators of severity (*i.e.* high MELD score, ICU admission, intubation, renal replacement therapy, mean arterial pressure, use of vasopressor amines, and volume of blood transfusion at Day 0), could discriminate subsequent use of rTIPS. In addition, the date and site of enrollment did not affect access to rTIPS, which was implanted in patients from the nine participating centers. Within the first 5 days, 12 patients in the ES group and eight patients in the BT group underwent rTIPS placement (including two bare stents in the BT group). There was no significant difference in rate of use of rTIPS between groups (ES: 19.1% *vs.* BT: 12.7%; *p* = 0.180), period (prospective enrollment or not: 38.7% *vs.* 25.0%, respectively; *p* = 0.242) or time taken to place the rTIPS (1.3 ± 0.5 days in the BT group *vs.* 1.9 ± 1.4 days in the ES group; *p* = 0.339). No TIPS was placed between Day 5 and Week 6.

### Mortality

The overall mortality rate on Day 5 was 42.9% (n = 27). Eight additional patients died between Day 5 and Week 6 (Table 3). Refractory hemorrhagic shock was the primary cause of mortality on Day 5 (79.2% of cases), whereas multiorgan failure accounted for 8.3% of deaths. The 6-week mortality rate was 55.6%. Recurrent bleeding was the cause of death in 37.5% of cases between Day 5 and Week 6, whereas sepsis was involved in 25.0% of cases and multiorgan failure in 37.5%.

**Table 3:** Outcome events.

Variable	BT	ES	Total	*p* values
Mortality at Week 6	24 (72.7%)	11 (36.7%)	35 (55.6%)	0.0015
Mortality at Day 5	21 (63.6%)	6 (20.0%)	27 (42.9%)	0.0005
Control of bleeding at Day 5	10 (30.3%)	21 (70.0%)	31 (49.2%)	0.002
Recurrent bleeding at Day 7∗	1/12 (8.3%)	2/23 (8.3%)	3/36 (8.3%)	1.000
Recurrent bleeding at Week 6∗	1/10 (10.0%)	2/23 (8.7%)	3/33 (9.1%)	0.905

**Device-related AEs**				
At least one device-related AE	9 (27.3%)	17 (56.7%)	26 (41.3%)	0.018
Esophageal rupture	2 (6.1%)	0 (0.0%)	2 (3.2%)	0.493
Inhalation pneumonia	5 (15.2%)	3 (10.0%)	8 (12.7%)	0.539
Placement-related esophageal injury	4 (12.1%)	7 (23.3%)	11 (17.5%)	0.242
Chest pain	2 (6.1%)	2 (6.7 %)	4 (6.4%)	0.921
Device migration	0 (0.0%)	8 (26.7%)	8 (12.7%)	0.0015
Early withdrawal	2 (6.1%)	2 (6.7%)	4 (6.4%)	0.921
Bleeding after removal	0/7 (0.0%)	4/11 (36.4%)	4/18 (22.2%)	0.070
Ischemia after removal	0/11 (0.0%)	3/22 (13.7%)	3/33 (9.1%)	0.199

**Other OEs**				
Hepatic encephalopathy	10 (30.3%)	17 (56.7%)	27 (42.9%)	0.035
Infection	8 (24.2%)	9 (30.0%)	17 (27.0%)	0.607
Hepatorenal syndrome	1 (3.3%)	7 (23.3%)	8 (13.3%)	0.023
Cardiac arrest	4 (12.1%)	0 (0.0%)	4 (6.4%)	0.115

Figures are given for 33 patients in the BT group and 30 in the ES group. If data are missing for some variables, the denominators are given. Statistical differences were assessed using Chi-squared or Fisher's exact test.

AE, adverse event; BT, balloon tamponade; ES, esophageal stent; OE, outcome event.

∗ Analysis excludes patients with uncontrolled bleeding on Day 5.

#### Impact of the tamponade device

**Fig. 3:**
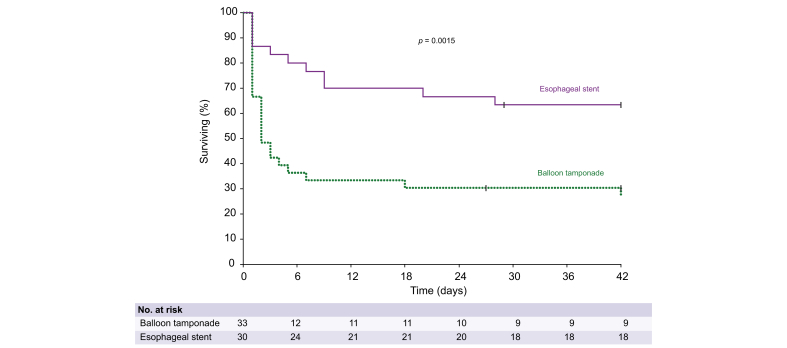
Survival up to 6 weeks according to the type of tamponade device used. Unadjusted overall survival at Day 42 (Week 6) was significantly higher in patients treated with an ES compared with PT (Log-rank test, *p* = 0.0015). This survival analysis shows a significant survival benefit in favor of ESs, with early and sustained divergence of the curves, emphasizing the impact of Ess on early mortality reduction. BT, balloon tamponade; ES, esophageal stent.

Six-week mortality was significantly lower in the ES group compared with the BT group (36.7% *vs.* 72.7%, respectively; *p* = 0.0015; Table 3 and Fig. 3; Figs S4 and S5), with a similar difference already present at Day 5 (20.0% *vs.* 63.6%, respectively; *p* = 0.0005). Early mortality within the first 2 days was also significantly lower in the ES group (13.3% *vs.* 51.2%, respectively, *p* = 0.0013). This survival difference remained significant in a sensitivity analysis restricted to the period during which ES was available (December 2010 to April 2023; Fig. S2). Time-dependent Cox models adjusted for MELD score, rTIPS, and stIPTW3 confirmed that ES was independently associated with improved 6-week survival (Table 4; Table S3). A landmark analysis restricted to patients alive on Day 2 yielded consistent results (Table S5B). When further restricted to patients alive on Day 5, the association was no longer statistically significant.

**Table 4:** Impact of ES on Week-6 mortality and early bleeding control across univariable and multivariable models.

Analysis	HR	95% CI	*p* values
**Impact of esophageal stent on Week-6 mortality**			
Univariable Cox regression	0.328	0.159–0.674	0.0024
Multivariable time-dependent Cox (ES + MELD∗ + rTIPS†) without PS-derived adjustment	0.229	0.105–0.498	0.0002
Multivariable time-dependent Cox (ES + MELD∗ + rTIPS^‡^ + stIPTW3 as covariate)	0.261	0.097–0.702	0.0078
Multivariable time-dependent Cox (ES+ MELD∗ + rTIPS^‡^) weighted by stIPTW3	0.269	0.119–0.605	0.0015

**Impact of ES on early bleeding control**			
Univariable analysis (Chi-squared)	5.367	1.827–15.763	0.0016
Multivariable logistic regression (ES + MELD∗ + rTIPS^‡^) without PS-derived adjustment	8.999	2.623–30.873	0.0005
Multivariable logistic regression (ES + MELD∗ + rTIPS^‡^ + stIPTW3∗ as covariate)	6.127	1.323–28.376	0.0205
Multivariable logistic regression (ES + MELD∗ + rTIPS^‡^) weighted by stIPTW3	14.716	3.145–68.865	0.0006

The use of ESs was consistently associated with improved outcomes in patients with refractory variceal bleeding. Across all models, their use resulted in a reduction in Week-6 mortality of at least 65%, and an ∼five fold increase in the likelihood of achieving early bleeding control compared with BT. The magnitude and consistency of these effects across univariable and multivariable models underscore the robustness of this therapeutic benefit.

BT, ballon tamponade; CI, confidence interval; ES, esophageal stent; HR, hazard ratio; IPTW, inverse probability of treatment (ES) weighting; MELD, model for end stage liver disease; OR, odds ratio; TIPS, transjugular portosystemic shunt.

∗ Expressed as continuous variables.

† rTIPS was included as a time-dependent variable in the Cox model.

#### Impact of rescue TIPS

Among patients who survived for 6 weeks, 47.4% (9/19) in the ES group and 66.7% (6/9) in the BT group had undergone rTIPS. Regardless of the tamponade device used, the 6-week mortality was significantly lower in patients who underwent rTIPS (25.5% *vs.* 67.5% in patients who did not undergo rTIPS; *p* = 0.0009, Fig S3A, S5). This association is further illustrated in Fig S3B which confirms the protective effect of rTIPS on 6-week survival in a landmark sensitivity analysis restricted to patients who survived the first 2 days. The Day 5 mortality was also significantly lower in patients who underwent rTIPS than in those who did not (10.0% *vs.* 58.1%, respectively; *p* = 0.0003). In the ES group, Day 5 and Week 6 mortality rates were 8.3% and 25.0%, respectively, with rTIPS, and 27.8% and 44.4%, respectively, without rTIPS (Log-rank test; *p* = 0.248). In the BT group, the mortality rates on Day 5 and Week 6 were 12.5% and 25.0%, respectively, with rTIPS and 80.0% and 84.0%, respectively, without rTIPS (Log-rank test; *p* = 0.0013).

#### Multivariable analysis

In the stIPTW3-adjusted time-dependent multivariable Cox model on mortality at Week 6, high MELD scores were associated with a significantly increased risk of death (hazard ratio [HR] = 1.056, 95% CI 1.018–1.094, *p* = 0.0035), whereas the use of ES (HR = 0.261; 95% CI 0.097–0.702, *p* = 0.0078) and rTIPS (HR = 0.211; 95% CI 0.059–0.752, *p* = 0.0165) were both found to be independent protective factors (Table S3). The robustness of these findings was tested in multivariable models weighted by stIPTW3 or incorporating stIPTW3 as a covariate, and multiple sensitivity analyses with alternative adjustment strategies (unadjusted, PS3-adjusted, adjusted with crude IPTW3, and stIPTW3 Cox models with no time-dependent covariates) (Table 4; Table S6). All models consistently supported the beneficial effects of ES on survival (Table 4).

### Bleeding

#### Control of initial bleeding

Control of initial bleeding was observed in 31 patients (49.2%), more frequently in the ES group than in the BT group (70.0% *vs.* 30.3%, respectively; *p* = 0.002; Table 3). Control of initial bleeding was also more frequent in the 20 patients who underwent rTIPS placement compared with those who did not (70.0% *vs.* 39.5%, respectively; *p* = 0.024). In patients who underwent rTIPS, control of initial bleeding did not differ between the ES and BT groups (75.0% *vs.* 62.5%, respectively; *p* = 0.550). Conversely, in the 43 patients who did not undergo rTIPS, bleeding control was observed more frequently in the ES group than in the BT group (66.7% *vs.* 20.0%, respectively; *p* = 0.002).

In the stIPTW3-adjusted logistic regression analysis, low MELD score (odds ratio [OR] = 1.088, 95% CI 1.031–1.148, *p* = 0.040), rTIPS (OR = 3.955, 95% CI 1.065–14.689, *p* = 0.0145), and ES (OR = 6.127, 95% CI 1.323–28.376, *p* = 0.0205) were independently associated with bleeding control at Day 5 (Table S4). Similar conclusions were reached in the stIPTW3-weighted logistic regression analysis, except for rTIPS, which had no independent effect (Table S4). Multivariable models that explored different adjustment strategies reinforced the robustness of the observed benefits of ES in achieving early bleeding control (Table 4, Table S6).

#### Recurrent bleeding

Of the 36 patients who survived to Day five (11 in the ES group with rTIPS, 13 in the ES group without rTIPS, seven in the BT group with rTIPS, and five in the BT group without rTIPS), two patients experienced recurrent bleeding on Day 7 in the ES group and one in the BT group. Two of these three had received rTIPS (one after ES, one after BT). Three additional episodes of recurrent bleeding were observed at Week 6. These episodes occurred in two patients in the ES group and one in the BT group. No significant differences were observed for recurrent bleeding between the groups.

### Other outcome events

Table 3 presents the outcomes observed throughout the follow-up period. No patient underwent liver transplantation within the first 6 weeks following the index variceal bleeding. With regard to device-related AEs, the available records indicate that four cases of esophageal bleeding and three cases of ischemia on stent removal were reported in the ES group. By contrast, no such complications occurred in the BT group. Two cases of esophageal rupture and eight cases of stent migration were observed in the BT and ES groups, respectively. The occurrence of esophageal ulcers, inhalation pneumonia, chest pain, and early withdrawal did not differ between the two groups. Overall, 17 patients in the ES group and nine patients in the BT group (56.7% *vs.* 27.3%, respectively; *p* = 0.018) experienced at least one device-related AE. Most of these events involved stent migration (Table 3) without clinical consequences. The occurrence of hepatic encephalopathy or hepatorenal syndrome at Week 6 was more frequent in the ES group than in the BT group (Table 3), but the differences were not significant when considering only patients alive at Day 5.

## Discussion

Refractory variceal bleeding, defined as bleeding that persists despite pharmacological and endoscopic treatments, is a rare but serious complication of cirrhosis. It accounts for an estimated 15–20% of variceal bleeding cases.^14^^,^^15^ Despite the use of rTIPS, mortality rates in different studies range from 20% to 50%.^5^^,^^12^ In this context, any contribution to improving survival would be welcome.

In this nationwide multicenter cohort study of patients with refractory variceal bleeding, self-expanding ESs were associated with significantly improved early bleeding control and reduced mortality on Day 5 and Week 6, compared with BT. To the best of our knowledge, this is the first comparative study to document the superiority of ES in this setting. These associations remained consistent following rigorous adjustment using stIPTW3, based on a well-performing PS model that included 23 pretreatment covariates. Notably, the 5-day mortality rate decreased from 63.6% with BT to 20.0% with ES, and the 6-week mortality rate decreased from 72.7% to 36.7%. This dramatic reduction in early mortality further underscores the protective role of ESs during the acute phase of bleeding (Fig. 3). The highest survival rates were observed in patients who received both ES and rTIPS, with 91.7% of patients alive at Day 5 and 75% alive at Week 6. The benefit of ES observed was not only statistically robust, but also clinically meaningful, especially considering the severity of illness in this cohort (MELD 22.1 and 73.0% Child-Pugh C). The impact on bleeding control persisted as a significant factor in fully adjusted models (OR = 6.127, *p* = 0.0205; Table 4; Table S4). In addition, the ES was found to be independently associated with decreased 6-week mortality (HR = 0.261, *p* = 0.0078; Table 4; Table S3). The survival benefit resulting from ES was noteworthy and somewhat unexpected in its magnitude. Despite the frequent use of rTIPS during the study period, which did not differ between groups, and the significant impact of liver failure, an unlikely influence of a tamponade device,^16^ the survival benefit associated with ES remained unaffected. rTIPS likewise provided protection in most cases, although its statistical significance varied depending on the modeling strategy. Taken together, these findings support a sequential strategy in which tamponade ensures immediate mechanical compression to stop bleeding, and rTIPS offers definitive treatment for portal hypertension. While the best outcomes were seen with a combination of ES and rTIPS, ES alone was also associated with improved survival.

The credibility of these results is bolstered by several factors. The cohort included patients from nine centers, encompassing the main configurations of care delivery, including university and non-university hospitals, with or without ICUs, and with or without local TIPS availability. Although it was not possible to formally confirm strict consecutive inclusion because of potential limitations in ICD-based retrospective identification, the pragmatic design and efforts made to include as many patients as possible through national hepatology networks suggest good representativeness. Thirty-one patients were prospectively enrolled in the study, and the remainder were included retrospectively based on consistent diagnostic coding. The duration of inclusion varied across centers. However, a centralized methodology and standardized data collection processes minimized heterogeneity. Two additional methodological strengths should be noted. First, the study was restricted to centers that had used both tamponade devices during the inclusion period, which limited the risk of center-level confounding. Second, rigorous eligibility criteria were implemented to ensure a homogeneous population (Fig. 1). Only patients with cirrhosis were included, and all patients with hepatocellular carcinoma were excluded because it can compromise the optimal management of variceal bleeding, particularly access to ICUs. Any patient with gastric varices, even if presumed non-bleeding, was also excluded to avoid introducing confusion in the analysis of esophageal compression efficacy on survival outcomes. The third PS model exhibited remarkable discrimination, as evidenced by an AUROC of 0.935 (Materials and methods S1B). In addition, substantial covariate balance was attained following the implementation of a weighting scheme. Sensitivity analyses using various adjustment methods (PS covariate, crude IPTW, and stlPTW3) consistently reproduced the effects of ES on both endpoints (Table S6).

However, the present study had several limitations. The study was observational in nature, and unmeasured confounding cannot be fully excluded despite the robust methodology used. The impact of rTIPS on survival appears to be less significant than that of ES, with varying levels of significance across multivariable models (Tables S3 and S4). Among the time-dependent Cox models, only the model that included stIPTW3 as a covariate showed a statistically significant protective effect of rTIPS. By contrast, the model weighted by stIPTW3 did not (Table S3). This discrepancy is likely the result of the temporal overlap between rTIPS timing and early mortality. Notably, 42.4% of deaths occurred within the first 2 days, during which >90% of rTIPS procedures were performed. Consequently, patients who died early were inherently excluded from the rTIPS group, thereby introducing immortal time bias in non-time-dependent analyses. Although time-dependent modeling addresses immortal time bias, it can yield unstable estimates in this context because of the high concentration of events within a narrow time window. This makes the analysis highly sensitive to minor imbalances between groups. Notably, all non-corrected models including unadjusted, IPTW-adjusted, and landmark analyses excluding early deaths, consistently demonstrated a survival benefit of rTIPS. The variability observed in the effect of rTIPS, as revealed by the analyses, is hypothesized to be driven primarily by analytic constraints rather than by true clinical inefficacy. Indeed, the protective effect of rTIPS was confirmed by a sensitivity analysis that excluded all patients who died before Day 2.

It is noteworthy that early deaths before Day 2 were considerably more prevalent in the BT group (51.2% *vs.* 13.3% in the ES group; *p* = 0.0013). However, the survival benefit associated with ES remained significant in the landmark analysis restricted to patients who survived at least 48 h (Table S5B). This finding reinforces the robustness of the ES effect and suggests that its protective association cannot be explained solely by a lead-time advantage or an imbalance in early deaths. Conversely, in analyses restricted to the prospectively enrolled subset, the beneficial effect of ES was no longer statistically significant, most likely because of insufficient power rather than an absence of effect.

In addition, there are certain contextual and methodological aspects that merit attention. First, access to ESs was initiated in December 2010 in a limited number of centers and subsequently became universal after 2021. This raises the possibility of a period effect (Fig. S1). However, the stIPTW3 model effectively neutralized differences in the year of inclusion and prospective enrollment between groups. This is evident from the standardized mean difference of <0.2 for both covariates (Fig. 2; Table S2. Furthermore, a sensitivity analysis restricted to the broader period from December 2010 to April 2023, corresponding to the period of potential ES availability, confirmed the observed benefit (Fig. S2 and Table S5A), thereby mitigating this concern. Second, the determination of causes of death was not centrally adjudicated, and decisions about rTIPS placement were not based on predefined criteria. This might indicate center-specific practices or implicit assessments of futility in critically ill patients,^17^ which could introduce selection bias. Third, a randomized controlled trial would be the most robust design for validating these findings; however, such an approach is unlikely to be feasible. Refractory variceal bleeding requiring tamponade is a rare occurrence, and the French ICD records estimated that only 1.4% of variceal bleeding cases per year involved tamponade.^18^ Furthermore, recent data from French and international registries indicate that tamponade was used in only 103 (3.4%) of 3,019 patients enrolled over 5 years in 87 European centers.^19^ The low incidence of the condition in question poses significant logistical and ethical challenges to implementing of large-scale randomized trials within a reasonable timeframe.

Another consideration is that ESs might create more favorable conditions for rTIPS placement by allowing for a longer tamponade duration and maintaining luminal patency. This was not directly measurable in the present study. This hypothesis is supported by the finding that an ES can remain in place for several days, ensuring hemodynamic stabilization and facilitating transfer to a tertiary care facility. Although the number of transfers was limited in this cohort, this effect might be underestimated because of the retrospective nature of some of the data. Consequently, the function of the ES might extend beyond achieving hemostasis immediately, thereby contributing to the success of subsequent therapeutic interventions.

In summary, the use of tamponade with a self-expanding stent should not be considered merely an interim measure while awaiting definitive therapy. Rather, it should be recognized as a crucial component of initial management. The substantial decrease in early mortality, especially when ES is used with timely rTIPS, supports a two-step therapeutic approach that could transform outcomes in this high-risk population. These findings should inform updates to international practice guidelines, including the Baveno Consensus, by recognizing the therapeutic value of ESs as integral interventions, rather than only bridges. Further prospective data are necessary to validate these findings and to optimize care pathways. This includes facilitating prompt access to specialized centers for TIPS placement.

## Abbreviations

AE, adverse event; APA, antiplatelet agent; BT, balloon tamponade; ES, esophageal stent; EV, esophageal varices; GI, gastrointestinal; HR, hazard ratio; ICD, International Classification of Diseases; ICU, intensive care unit; IPTW, inverse probability of treatment weighting; MAP, mean arterial pressure; MASLD, metabolic dysfunction-associated steatotic liver disease; MELD, model for end-stage liver disease; OE, outcome event; OR, odds ratio; PH, portal hypertension; PS, propensity score; pTIPS, pre-emptive transjugular intrahepatic portosystemic shunt; RBC, red blood cell; rTIPS, rescue transjugular intrahepatic portosystemic shunt; SMD, standardized mean difference; stIPTW3, stabilized and truncated IPTW; TIPS, transjugular intrahepatic portosystemic shunt.

## Financial support

No funding was received for this study.

## Authors’ contributions

Study conceptualization: DW, J-PC, SK, J-PA, MC-D, MG, MR. Methodology and questionnaire: DW, J-PC, MC, MR. Investigation and data collection: MC, CB, AR, GB, IO-H, NR, CL, CR, MC-D, LC, A-JR, LE, GC, FW, AG, EB-J, J-PA. Data curation and statistical analysis: VDM, DW. Drafting of manuscript: VDM, DW. Review and editing: DW, MC, CB, J-PC, AR, GB, IO-H, NR, CL, CR, MC-D, LC, A-JR, LE, GC, FW, AG, MG, EB-J, SK, J-PA, MR, and VDM. All authors approved the final version of the manuscript. Guarantor of manuscript: DW.

## Data availability

Data collected for the study, including individual participant data, will be made available under transfer agreement from the corresponding author upon reasonable request. A signed data access agreement is required. The data provided will de-identify the participant data. There are restrictions on how the data can be used or how long the data will be available.

## Conflicts of interest

The authors have no conflicts of interest to report.

Please refer to the accompanying ICMJE disclosure forms for further details.

## References

[1] Gralnek I.M., Garcia-Pagan J.C., Gea V.H. (2024). Challenges in the management of esophagogastric varices and variceal hemorrhage in cirrhosis - a narrative review. Am J Med.

[2] Kaplan D.E., Ripoll C., Thiele M. (2024). AASLD Practice Guidance on risk stratification and management of portal hypertension and varices in cirrhosis. Hepatology.

[3] Sengstaken R.W., Blakemore A.H. (1950). Balloon tamponage for the control of hemorrhage from esophageal varices. Ann Surg.

[4] Hunt P.S., Korman M.G., Hansky J. (1982). An 8-year prospective experience with balloon tamponade in emergency control of bleeding esophageal varices. Dig Dis Sci.

[5] Keung C.Y., Morgan A., Le S.T. (2022). Survival outcomes and predictors of mortality, re-bleeding and complications for acute severe variceal bleeding requiring balloon tamponade. World J Hepatol.

[6] Zehetner J., Shamiyeh A., Wayand W. (2008). Results of a new method to stop acute bleeding from esophageal varices: implantation of a self-expanding stent. Surg Endosc.

[7] Pfisterer N., Riedl F., Pachofszky T. (2019). Outcomes after placement of a SX-ELLA oesophageal stent for refractory variceal bleeding-a national multicentre study. Liver Int.

[8] Lo G.H. (2017). The use of esophageal stent in controlling acute refractory variceal bleeding. Hepatology.

[9] Songtanin B., Kahathuduwa C., Nugent K. (2024). Esophageal stent in acute refractory variceal bleeding: a systematic review and a meta-analysis. J Clin Med.

[10] de Franchis R., Bosch J., Garcia-Tsao G. (2022). Baveno VII - renewing consensus in portal hypertension. J Hepatol.

[11] Escorsell A., Pavel O., Cardenas A. (2016). Esophageal balloon tamponade *vs.* esophageal stent in controlling acute refractory variceal bleeding: a multicenter randomized, controlled trial. Hepatology.

[12] Rudler M. (2022).

[13] Garcia-Pagan J.C., Caca K., Bureau C. (2010). Early use of TIPS in patients with cirrhosis and variceal bleeding. N Engl J Med.

[14] Rodrigues S.G., Cardenas A., Escorsell A. (2019). Balloon tamponade and esophageal stenting for esophageal variceal bleeding in cirrhosis: a systematic review and meta-analysis. Semin Liver Dis.

[15] Kumar R., Kerbert A.J.C., Sheikh M.F. (2021). Determinants of mortality in patients with cirrhosis and uncontrolled variceal bleeding. J Hepatol.

[16] de Franchis R., Baveno V.I.F. (2015). Expanding consensus in portal hypertension: report of the Baveno VI Consensus Workshop: stratifying risk and individualizing care for portal hypertension. J Hepatol.

[17] Thabut D., Pauwels A., Carbonell N. (2017). Cirrhotic patients with portal hypertension-related bleeding and an indication for early-TIPS: a large multicentre audit with real-life results. J Hepatol.

[18] Haute Autorité de Santé Removable covered self-expanding esophageal stent (SX-ELLA Stent Danis) and its extraction system (ELLA Extractor). http://www.has-sante.fr/jcms/p_3211567/fr/danis-stent.

[19] Weil D., Thabut D., Hernandez-Gea V. (2022). Rescue TIPS (rTIPS) must be considered as soon as a tamponade is used: results from two international multicenter cohorts of 3019 patients with portal hypertension (PHT)-related bleeding. Hepatology.

