# Supplementary material for: Improved survival in patients with refractory variceal bleeding treated with esophageal stents: A multicenter cohort study
Source: JHEP Rep. 2025 Aug 30;7(12):101581. doi: 10.1016/j.jhepr.2025.101581 (PMC12657723; doi:10.1016/j.jhepr.2025.101581)
Supplement: Multimedia component 1 [file mmc1.pdf]

Improved survival in patients with refractory variceal bleeding treated with esophageal stents: A multicenter cohort study

Delphine Weil, Morgane Clément, Charlotte Bouzbib, Jean-Paul Cervoni, Andrimalala Raoto, Grégoire Boivineau, Isabelle Ollivier-Hourmand, Noémi Reboux, Caroline Lemaitre, Cassandra Rayer, Marine Camus-Duboc, Ludovic Caillo, André-Jean Remy, Laure Elkrief, Guillaume Conroy, Faustine Wartel, Armand Garioud, Maeva Guillaume, Edouard Bardou-Jacquet, Stéphane Koch, Jean-Pierre Arpurt, Marika Rudler, Vincent Di Martino, ANGH, CREGG, SFED, GRAPHE, CFHTP groups

Table of contents

Supplementary material 1 2

Table S1 2

Table S2 4

Fig. S1 5

Fig. S2 6

Fig. S3 7

Fig. S4 8

Fig. S5 9

Table S3 10

Table S4 11

Table S5 12

Table S6 13

Supplementary material 1: Construction and Implementation of the Propensity Score Model for Treatment Effect Adjustment

S1A: Development and Selection of the Propensity Score Model

Given the observational design of the study and baseline heterogeneity between treatment groups, we constructed multiple propensity score (PS) models to adjust for confounding in the comparison of outcomes between patients treated with esophageal stents (ES) and those receiving balloon tamponade (BT). Eleven candidate models (PS1 to PS11) were developed using logistic regression, each incorporating different combinations of up to 36 baseline covariates.

To ensure robustness, only variables available prior to treatment initiation were included, while those showing multicollinearity or clinical redundancy were excluded. Model performance was assessed based on two key metrics of covariate balance after weighting: (i) the mean standardized mean difference (SMD) across all covariates, and (ii) the number of covariates achieving an SMD below the accepted threshold of 0.2.

Inverse probability of treatment weighting (IPTW) was computed as 1/PS for treated patients and 1/(1–PS) for controls. Stabilized weights were calculated using the marginal probability of treatment, and all weights were truncated at a maximum of 10 to minimize variance inflation. Among all models tested, PS3 demonstrated the best overall balance, with a mean SMD of 0.453 and 23 out of 36 covariates adequately balanced after applying stabilized and truncated IPTW (stIPTW). These results are summarized in the table below.

Table S1

PS Model	Number of included variables	AUROC	95% CI AUROC	Mean SMD (crude IPTW)	Number of Variables with SMD < 0.2 (crude IPTW)	Mean SMD (stabilized & truncated IPTW)	Number of variables with SMD < 0.2 (stabilized & truncated IPTW)
**PS1**	18	0.873	[0.766 – 0.953]	0.634	12	0.610	14
**PS2**	22	0.909	[0.832 – 0.969]	2.442	2	0.476	21
**PS3**	23	0.935	[0.865 – 0.979]	2.212	1	**0.453**	**23**
**PS4**	24	0.944	[0.885 – 0.983]	1.994	1	0.455	23
**PS5**	23	0.923	[0.851 – 0.978]	2.315	2	0.468	22
**PS6**	22	0.907	[0.827 – 0.969]	2.665	1	0.497	20
**PS7**	23	0.938	[0.868 – 0.982]	2.064	3	0.458	22
**PS8**	21	0.888	[0.795 – 0.960]	3.07	1	0.510	20
**PS9**	20	0.841	[0.742 – 0.927]	3.027	2	0.514	24
**PS10**	21	0.846	[0.738 – 0.932]	2.868	2	0.517	23
**PS11**	22	0.877	[0.779 – 0.955]	2.635	4	0.501	23

S1B: Specification and Predictive Performance of PS3

The propensity score for receiving esophageal stenting was estimated using a multivariable logistic regression model (PS3) including 23 covariates. The logit of the PS3 was calculated as:

Logit(PS3) *= 0.1809 ×* ***Age*** *− 11.3146 × (****General anesthesia*** *= 1) + 0.2967 ×* ***Albumin*** *− 0.0157 ×* ***Year of inclusion*** *− 1.4443 × (****Ascites*** *= 1) − 1.5522 × (****History of portal hypertension-related bleeding*** *= 1) + 1.7964 × (****Known esophageal varices*** *= 1) − 4.8057 × (****Metabolic cirrhosis*** *= 1) − 8.3885 × (****University hospital*** *= 1) + 0.0212 ×* ***Creatinine*** *+ 1.0677 ×* ***Time from bleeding onset to tamponade*** *− 1.5524 × (****Hepatic encephalopathy*** *= 1) − 0.6684 ×* ***Hemoglobin*** *+ 11.5724 × (****Intubation*** *= 1) − 2.1158 × (****Vasopressors*** *= 1) − 0.1420 ×* ***MELD score*** *+ 0.1602 ×* ***RBC units transfused on day 0*** *+ 0.0788 ×* ***Mean arterial pressure*** *+ 2.8440 × (****ICU admission*** *= 1) + 3.7778 × (****Prospective enrollment*** *= 1) + 2.1347 ×* ***Child-Pugh score*** *− 0.2768 × (****Male sex*** *= 1) − 1.4305 × (****Beta-blocker treatment*** *= 1).*

The individual probability (PS) was then obtained by applying the standard logistic transformation: PS = exp(Logit) / [1 + exp(Logit)].

The figure below illustrates the receiver operating characteristic (ROC) curve showing the discriminative ability of the PS3 model to predict the use of esophageal stenting. The model demonstrated excellent performance, with an area under the ROC curve (AUROC) of 0.935 (95% CI: 0.876–0.981), confirming its strong ability to distinguish between patients who received esophageal stenting and those treated with balloon tamponade.

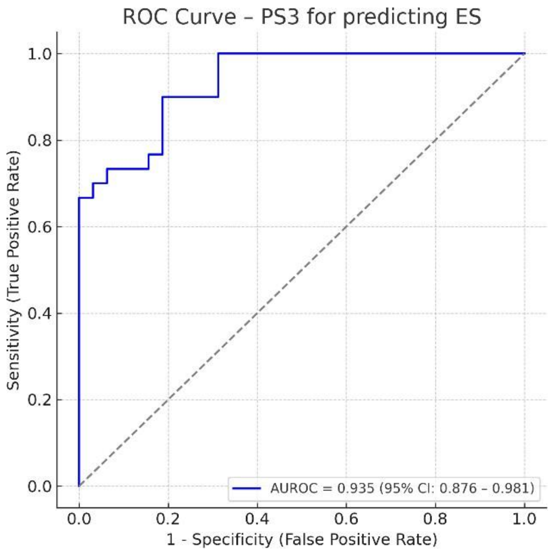

S1C: Weighting and Adjustment Strategy

To evaluate the effectiveness of the weighting strategy derived from the PS3 model, we assessed the standardized mean differences (SMDs) for key baseline covariates before and after weighting. The table below presents SMDs in the unweighted sample, after crude IPTW (IPTW3), and after application of stabilized and truncated IPTW (stIPTW3), with weights capped at 10. While crude IPTW sometimes exacerbated imbalance—with several covariates exceeding the conventional SMD threshold of 0.5—stabilized and truncated IPTW3 consistently improved covariate balance across all variables. Most SMDs fell below 0.2, indicating satisfactory adjustment and supporting the use of stIPTW3 for all subsequent outcome analyses.

Table S2.

Variable	SMD unweighted	SMD with crude IPTW3	SMD with stabilized and truncated PTW3
Hepatic encephalopathy	0,008	1,323	0,003
Melena	0,125	1,000	0,032
Alcoholic cirrhosis	0,021	0,440	0,037
Nb of RBC packs transfused	0,041	2,482	0,058
MASLD-related cirrhosis	0,039	0,796	0,058
History of Hepatic encephalopathy	0,203	0,190	0,068
Ascites	0,144	1,051	0,071
APAs	0,017	0,693	0,073
Pugh score	0,375	1,697	0,079
Hematemesis	0,112	1,083	0,079
Age	0,014	0,510	0,088
Male	0,079	0,459	0,090
Transfusion	0,021	0,695	0,104
Known EVs	0,214	0,572	0,109
Prospective enrollment	0,517	1,390	0,110
Vasopressors	0,000	1,073	0,116
ICU admission	0,134	1,031	0,116
Pharmacologic Treatment of variceal bleeding	0,100	0,701	0,125
Intubation	0,357	0,749	0,140
ß-Blockers	0,256	1,159	0,142
Year of enrollment	0,596	0,856	0,151
Albumin	0,089	2,526	0,165
TIPS prior index bleeding	0,372	1,734	0,174
History of PTH-related GI bleeding	0,129	0,464	0,181
General Anesthesia	0,428	1,230	0,218
MAP	0,220	1,470	0,221
Prothrombin time	0,314	1,285	0,242
Hemoglobin	0,507	0,300	0,263
Bilirubin	0,202	0,748	0,264
Diuretics	0,256	1,484	0,289
Rectorrhagia	0,164	0,308	0,345
Creatinine	0,181	0,565	0,406
Endoscopy on day 0	0,582	0,281	0,475
MELD score	0,361	1,651	0,480
University Hospital	0,771	0,776	0,564
History of ascites	0,448	1,438	0,604

Abbreviations: APAs: Anti-platelet agents; EVs: esophageal varices; GI: gastro-intestinal; ICU: intensive care unit; MAP: mean arterial pressure; MASLD: Metabolic dysfunction–Associated Steatotic Liver Disease; MELD: model for end-stage liver disease; PHT: portal hypertension; TIPS: transjugular intrahepatic portosystemic shunt; RBC: red blood cell.

Fig. S1: Patient inclusion timeline by center and treatment

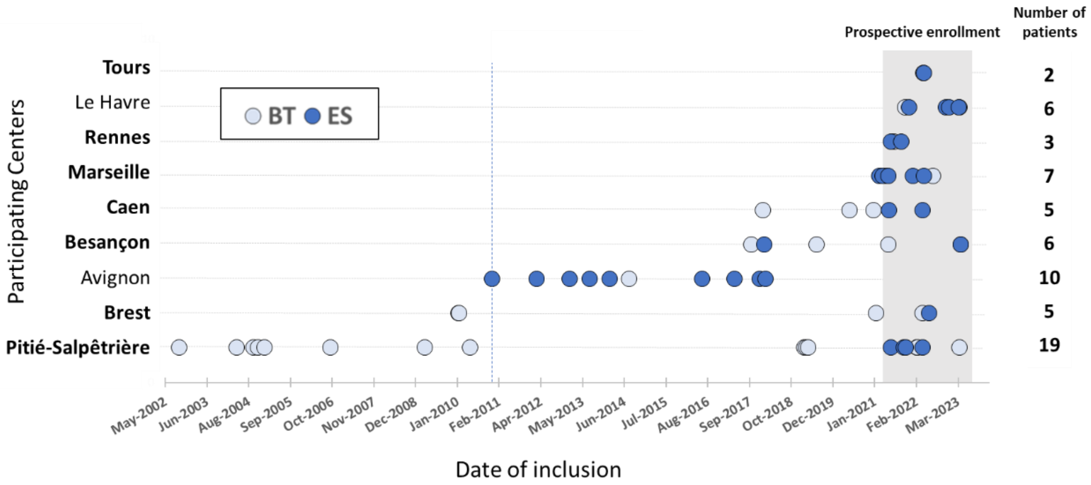

Each dot represents a patient included in the study, positioned according to the date of inclusion (X-axis) and the participating center (Y-axis). All centers used both Balloon Tamponade (BT, light blue dots) and Esophageal Stent (ES, dark blue dots). The vertical dashed line indicates the beginning of the period during which ES was available. Centers affiliated with university hospitals are shown in bold. The grey-shaded area indicates the period during which inclusions were conducted prospectively. The total number of patients included in each center is shown in the right-hand column.

Fig. S2: Week-6 survival in the ES and BT groups. Sensitivity analysis restricted to the period from December 2010 to April 2023.

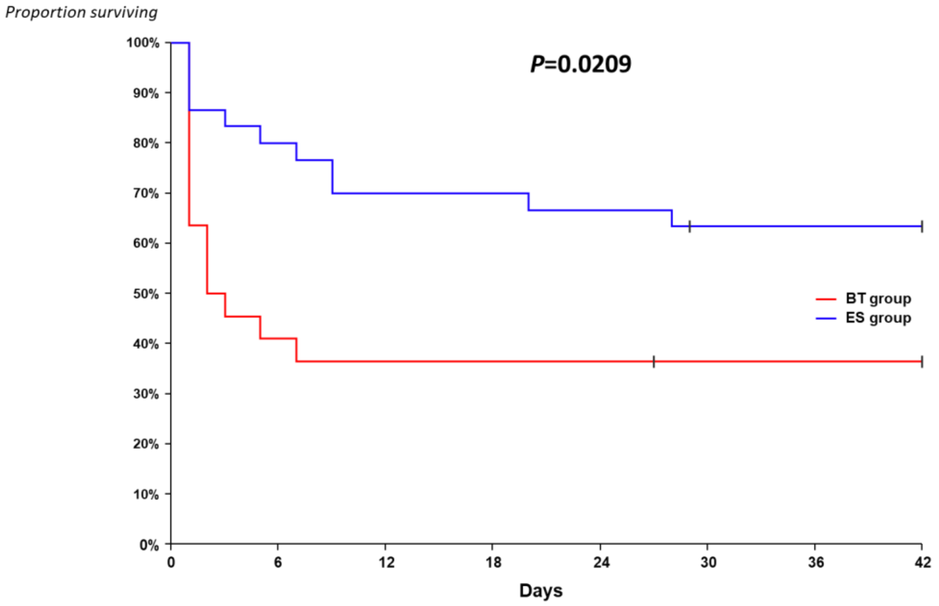

Sensitivity analysis restricted to the period from December 2010 to April 2023, during which esophageal stenting was progressively introduced across centers. Only the 53 patients included during periods and in centers where ES was available were analyzed. Six-week survival remained significantly higher in the ES group (Log-rank test, p= 0.0209), supporting the robustness of the association.

Fig. S3: Impact of rescue transjugular intrahepatic portosystemic shunt (rTIPS) on week-6 survival

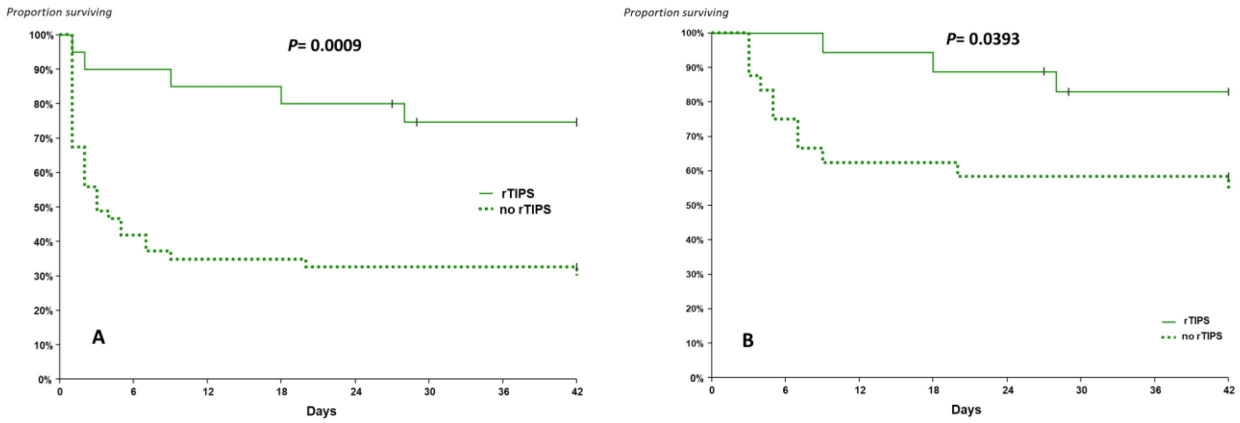

These figures illustrate the protective impact of rTIPS on week-6 mortality.

A: Analysis of the entire study population.

B: Landmark sensitivity analysis to minimize immortal time bias related to TIPS. Because 90% of rescue TIPS were performed within the first two days, this analysis excluded all patients who died before day 2. The solid green line indicates patients who underwent rTIPS, whereas the dotted green line indicates patients who did not undergo rTIPS. The p-values are derived from log-rank tests.

Fig. S4: Comparison of week-6 mortality between ES and BT groups among subgroups

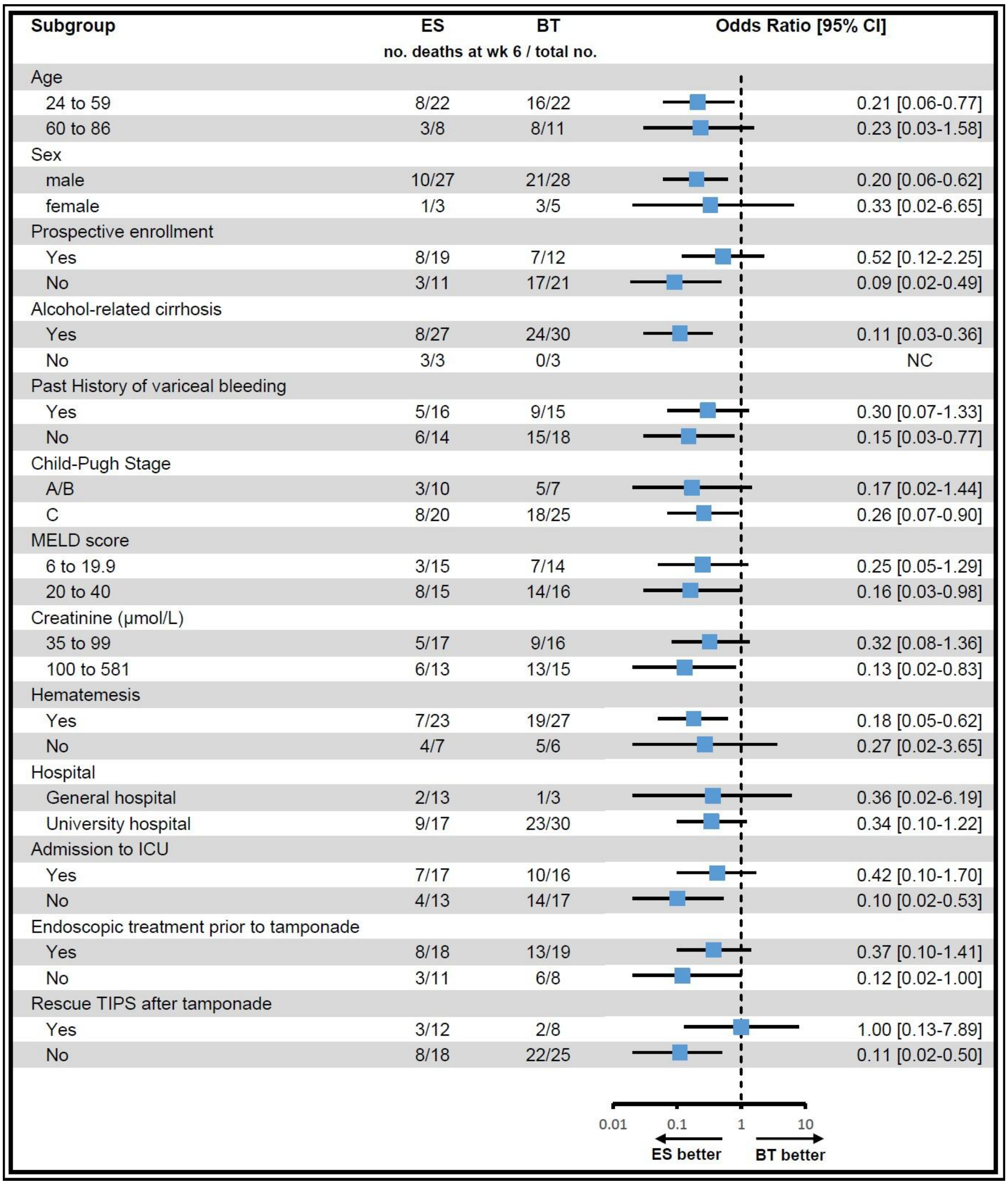

Subgroup	ES	BT	Odds Ratio [95% CI]
	no. deaths at wk 6 / total no.		
Age			
24 to 59	8/22	16/22	0.21 [0.06-0.77]
60 to 86	3/8	8/11	0.23 [0.03-1.58]
Sex			
male	10/27	21/28	0.20 [0.06-0.62]
female	1/3	3/5	0.33 [0.02-6.65]
Prospective enrollment			
Yes	8/19	7/12	0.52 [0.12-2.25]
No	3/11	17/21	0.09 [0.02-0.49]
Alcohol-related cirrhosis			
Yes	8/27	24/30	0.11 [0.03-0.36]
No	3/3	0/3	NC
Past History of variceal bleeding			
Yes	5/16	9/15	0.30 [0.07-1.33]
No	6/14	15/18	0.15 [0.03-0.77]
Child-Pugh Stage			
A/B	3/10	5/7	0.17 [0.02-1.44]
C	8/20	18/25	0.26 [0.07-0.90]
MELD score			
6 to 19.9	3/15	7/14	0.25 [0.05-1.29]
20 to 40	8/15	14/16	0.16 [0.03-0.98]
Creatinine (µmol/L)			
35 to 99	5/17	9/16	0.32 [0.08-1.36]
100 to 581	6/13	13/15	0.13 [0.02-0.83]
Hematemesis			
Yes	7/23	19/27	0.18 [0.05-0.62]
No	4/7	5/6	0.27 [0.02-3.65]
Hospital			
General hospital	2/13	1/3	0.36 [0.02-6.19]
University hospital	9/17	23/30	0.34 [0.10-1.22]
Admission to ICU			
Yes	7/17	10/16	0.42 [0.10-1.70]
No	4/13	14/17	0.10 [0.02-0.53]
Endoscopic treatment prior to tamponade			
Yes	8/18	13/19	0.37 [0.10-1.41]
No	3/11	6/8	0.12 [0.02-1.00]
Rescue TIPS after tamponade			
Yes	3/12	2/8	1.00 [0.13-7.89]
No	8/18	22/25	0.11 [0.02-0.50]

Subgroup analyses of week-6 mortality comparing esophageal stenting (ES) and balloon tamponade (BT). Odds ratios favoring ES were consistently observed across most subgroups, despite small sample sizes. The protective effect of ES was particularly marked in patients aged <60, with alcohol-related cirrhosis, high MELD or creatinine levels, and in those not admitted to the intensive care unit (ICU). In patients not receiving rescue transjugular intrahepatic portosystemic shunt (TIPS), ES was significantly associated with lower mortality, whereas no benefit was seen in those who subsequently received TIPS, suggesting that the survival advantage of ES is mainly observed when TIPS is not performed. Some confidence intervals are wide, reflecting limited power, but overall results support the robustness of ES efficacy across clinically relevant subgroups. The univariate analyses were performed with Chi-square tests.

Fig. S5: Respective impact of esophageal stenting (vs. balloon tamponade) and rescue TIPS on week-6 mortality

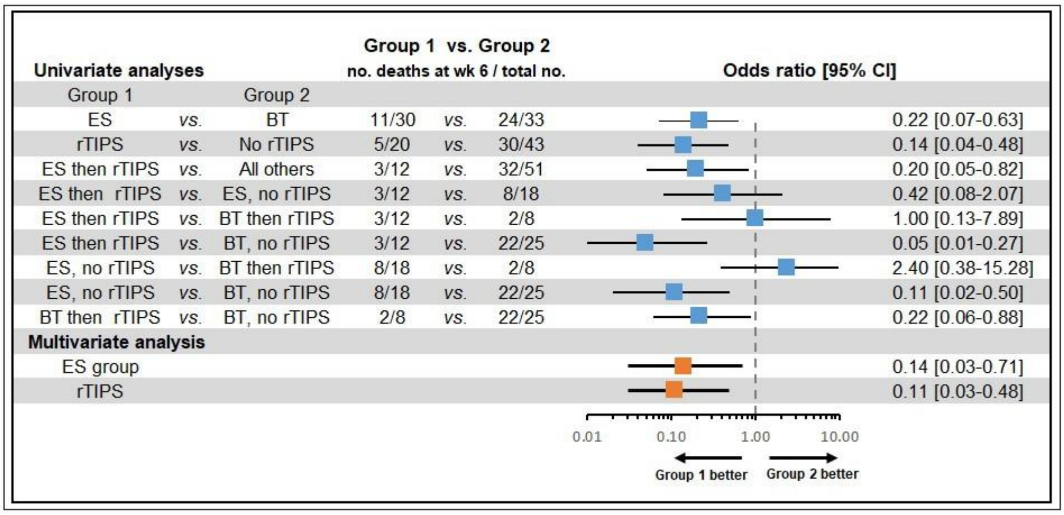

Multivariable logistic regression adjusted for MELD score and stabilized and truncated inverse probability of treatment weighting (stIPTW) (orange markers, lines 10 and 11) confirmed that both esophageal stenting (ES) and rescue TIPS (rTIPS) independently reduce six-week mortality. Despite limited subgroup sizes, two consistent findings emerged: ES was significantly more protective than BT in the absence of rTIPS (line 8), and rTIPS was associated with improved survival across all subgroups, including those initially managed with BT (lines 2, 4, 6, 7, and 9). Once rTIPS was placed, the additional benefit of prior ES was no longer apparent (line 5). The absence of a significant difference between patients treated with rTIPS and those who received ES without rTIPS (lines 4 and 7) likely reflects a type II error, and should not lead us to mistakenly conclude that early esophageal stenting obviates the need for timely rescue TIPS. The univariable analyses used Chi-squared tests.

Table S3: Multivariable time-dependent Cox regression analysis of Factors Associated with week-6 mortality

Model 63 patients; 35 events. r^2 (model) = 0.339.

Variables	HR	95%CI HR	r^2	p
**Esophageal Stent**	0.261	0.097 – 0.702	0.333	0.0078
**Rescue TIPS**	0.211	0.059 – 0.752	0.350	0.0165
**MELD score***	1.056	1.018 – 1.094	0.313	0.0035
**stIPTW3***	0.978	0.880 – 1.086	0.011	0.6719

TIPS : transjugular portosystemic shunt; MELD: model for end stage liver disease; HR: hazard ratio; CI: confidence interval; r^2: variance explained. stIPTW: stabilized and truncated Inverse probability of Treatment (Esophageal Stent) Weighting (*expressed as continuous variables).

TIPS was modeled as a time-dependent covariate to mitigate immortal time bias. Esophageal stent use and TIPS were both independently associated with reduced 6-week mortality, as well as low MELD scores. Model performance: concordance = 0.757; pseudo-R^2 = 0.339.

Mutivariable time-dependent Cox regression model weighted by stIPTW3 (instead of incorporating stIPTW3 as covariate) showed similar results for Esophageal Stent (HR = 0.269; 95%CI: 0.119-0.605; p = 0.0015) and MELD score (HR = 1.060; 95%CI: 1.011-1.110; p = 0.0129), but the effect of rescue TIPS was no longer significant (HR = 0.274; 95%CI: 0.056-1.349;p = 0.1120).

Table S4: Multivariable Logistic Regression Analyses of Factors Associated with Successful Bleeding Control at Day 5.

R^2 (model) = 0.333

Variables	OR	95%CI OR	p
**Esophageal Stent**	6.127	1.323– 28.376	0.0205
**Rescue TIPS**	3.955	1.065 – 14.689	0.0145
**MELD score**	0.919	0.871 – 0.970	0.0400
**stIPTW3**	1.076	0.898 – 1.288	0.4270

Mutivariable Logistic regression model weighted by stIPTW3 (instead of incorporating stIPTW3 as covariate) showed similar results for Esophageal Stent (HR=14.716; 95%CI: 3.145-68.865; p=0.0006) and MELD score (HR=0.920; 95%CI: 0.871-0.971; p=0.0027), but the effect of TIPS was no longer significant (HR=2.553; 95%CI: 0.468-13.910; p=0.2786).

Table S5: Sensitivity analyses on week-6 mortality: assessing the robustness of the esophageal stenting effect and correcting for TIPS-related immortal time bias

S5A: Time-dependent Cox multivariable model of week-6 mortality in subjects registered from December 2010 to April 2023 (time frame during which the esophageal stent was available).

Variables	HR	95%CI HR	p
**Esophageal Stent**	0.238	0.081 – 0.694	0.0086
**Rescue TIPS**	0.166	0.035 – 0.794	0.0217
**MELD score***	0.919	0.871 – 0.970	0.0400
**stIPTW3**	0.997	0.877 – 1.133	0.9583

52 patients ; 25 events ; R^2(model) = 0.235

TIPS was modeled as a time-dependent variable using a start–stop Cox model to accurately account for the time at risk before TIPS placement and prevent overestimation of its protective effect. The analysis covers patients who were enrolled during the period when the esophageal stent was available, either after its commercial release or before, at pilot centers.

S5B: Cox model for week-6 mortality restricted to the 42 patients who survived two days

Variables	HR	95%CI HR	p
**Esophageal Stent**	0.206	0.059 – 0.720	0.0134
**Rescue TIPS**	0.235	0.061 – 0.901	0.0347
**MELD score***	1.096	1.032 – 1.163	0.0028

42 patients; 14 events; R^2(Model) = 0.306

To account for potential residual immortal time bias—arising from early deaths that inherently preclude TIPS placement—we performed a landmark analysis restricted to patients who were still alive on day 2. This time point was selected because 90% of rescue TIPS procedures were performed within the first 48 hours after the bleeding episode. Restricting the cohort in this way ensures that all included patients had a real and comparable opportunity to receive the intervention, thus enhancing the validity of comparisons between the TIPS and no-TIPS groups. Given the limited number of events, only the model without adjustment for stIPTW3 is reported here. When this fourth covariate was included, the model converged but attenuated the statistical significance of the ES variable (p = 0.0501), despite a similar effect size (HR = 0.228).

Table S6: Multivariable sensitivity analyses for robustness assessment of the effect of esophageal stenting on early bleeding control and week-6 mortality: modifications of the adjustment covariate.

Logistic regression models for Control of initial bleeding	OR	95%CI OR	p
unadjusted model			
ES	8.999	2.626-30.872	0.0005
TIPS	4.016	1.085-14.863	0.0373
MELD score (continuous)	0.931	0.892-0.971	0.0009
model adjusted on propensity score			
ES	6.412	1.548-26.561	0.0104
TIPS	3.381	0.872-13.107	0.0781
MELD score (continuous)	0.913	0.860-0.970	0.0033
PS3	2.433	0.363-16.326	0.3599
model adjusted on crude IPTW			
ES	9.736	2.541-37.300	0.0009
TIPS	3.737	0.998-13.986	0.0503
MELD score (continuous)	0.932	0.894-0.972	0.0011
cIPTW3	1.000	0.608-1.645	1.0000
model adjusted on stabilized and truncated IPTW			
ES	6.127	1.323-28.376	0.0205
TIPS	3.955	1.065-14.689	0.04
MELD score (continuous)	0.919	0.871-0.970	0.0023
stIPTW3	1.076	0.898-1.288	0.4271

Cox models for 6-week mortality	HR	95%CI HR	p	r^2
Unadjusted model				
ES	0.254	0.117-0.551	0.0005	0.275
TIPS	0.195	0.067-0.565	0.0026	0.180
MELD score (continuous)	1.049	1.012-1.088	0.0083	0.222
Model adjusted on the propensity score				
ES	0.291	0.116-0.728	0.0083	0.185
TIPS	0.207	0.070-0.616	0.0046	0.207
MELD score (continuous)	1.052	1.013-1.092	0.0078	0.187
PS3	0.716	0.231-2.217	0.5624	0.011
Model adjusted on the crude IPTW				
ES	0.239	0.107-0.536	0.0005	0.283
TIPS	0.197	0.068-0.574	0.0029	0.224
MELD score (continuous)	1.049	1.012-1.088	0.0093	0.181
cIPTW3	1,000	1.000-1.000	0.4774	0.016
Model adjusted on the stabilized and truncated IPTW				
ES	0.276	0.102-0.748	0.0114	0.173
TIPS	0.195	0.067-0.567	0.0027	0.227
MELD score (continuous)	1.049	1.012-1.087	0.0094	0.18
stIPTW3	0.986	0.898-1.095	0.7954	0.002

To assess the robustness of our findings, we constructed multivariable models for the two primary endpoints—bleeding control on day 5 and week-6 mortality—using four adjustment strategies: unadjusted; adjusted for the propensity score (PS3); weighted using crude IPTW (cIPTW3); and weighted using stabilized and truncated IPTW (stIPTW3). The models included the following covariates: tamponade modality (esophageal stent [ES] vs. balloon tamponade [BT]), rescue TIPS, MELD score, and the adjustment variable (PS or IPTW).

The benefit of ES for bleeding control was strong and consistent. The unadjusted odds ratio (OR) was 8.999 (95% CI: 2.626–30.872, p = 0.0005), and remained significant after full adjustment with stIPTW3 (OR = 5.981, 95% CI: 1.624–22.024, p = 0.0073), indicating a robust independent effect of ES on early bleeding control. MELD score was inversely associated with bleeding control across all models. The effect of rescue TIPS was more variable: significant in the unadjusted and stIPTW3 models, but not in models adjusted for PS3 or using cIPTW3. This suggests that poorly specified adjustments may obscure true effects, either by increasing imbalance (as seen with cIPTW) or failing to address residual confounding (as with PS adjustment). In contrast, stIPTW3 restored both covariate balance and interpretability, revealing the protective effect of rescue TIPS otherwise masked by indication bias.

For week-6 mortality, results were more consistent. ES remained protective across all models (unadjusted HR=0.254, 95% CI: 0.117–0.551, p = 0.0005; stIPTW3-adjusted HR=0.276, 95% CI: 0.102–0.748, p = 0.0114). Rescue TIPS was also independently associated with improved survival in all configurations, including the fully adjusted model (HR = 0.195, 95% CI: 0.067–0.567, p = 0.0027). MELD remained a consistent predictor of mortality.

Importantly, all Cox models were non–time-dependent and did not account for the immortal time bias introduced by delayed TIPS. Since 90% of procedures occurred within 48 hours, most patients who died before day 2 could not receive TIPS. By not correcting for this bias, the analyses intentionally overestimated the apparent benefit of TIPS, thereby placing the initial tamponade modality (i.e., ES or BT) at a comparative disadvantage. The persistence of a significant association between esophageal stenting and improved week-6 survival under these analytically conservative conditions further reinforces the robustness of the observed benefit of esophageal stents.
