# Supplementary material for: Improved survival in patients with refractory variceal bleeding treated with esophageal stents: A multicenter cohort study
Source: JHEP Rep. 2025 Aug 30;7(12):101581. doi: 10.1016/j.jhepr.2025.101581 (PMC12657723; doi:10.1016/j.jhepr.2025.101581)
Supplement: Multimedia component 4 [file mmc4.pdf]

Improved survival in patients with refractory variceal bleeding treated with esophageal stents: A multicenter cohort study

Authors

Delphine Weil, Morgane Clément, Charlotte Bouzbib, …, Jean-Pierre Arpurt, Marika Rudler, Vincent Di Martino

Correspondence

dweil@chu-besancon.fr (D. Weil).

Graphical abstract

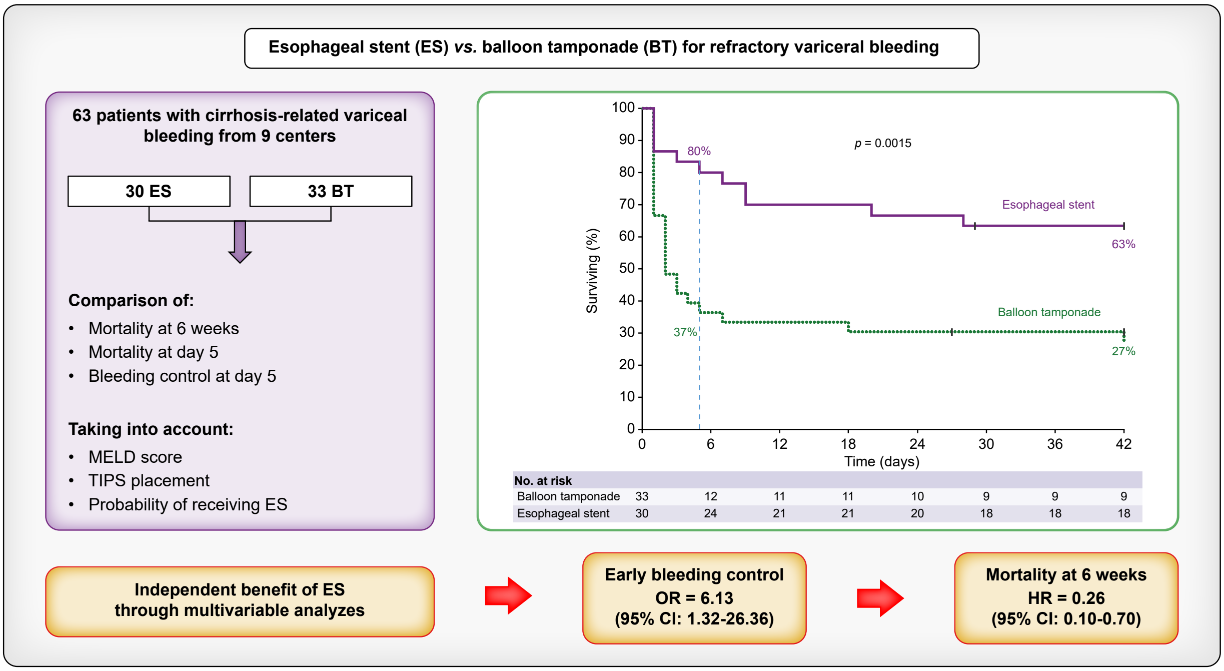

Highlights:

- After tamponade, all-cause mortality was 42.9% at Day 5 and 55.6% at week six.
- Both ES (rather than BT) and rTIPS improved bleeding control at day five.
- Six-week mortality was lower with ES *vs.* BT and in patients who underwent rTIPS.
- ES plus rTIPS provided the highest survival rate (91.7% at day five; 75.0% at week six).

Impact and implications:

Tamponade devices are crucial in the management of refractory variceal bleeding, with balloon probes being the only option until the early 2000s. More recently, self-expanding ESs have been recommended, although their superiority over BT in terms of survival has not yet been demonstrated. Our multicenter cohort study, using propensity score analysis, found that ESs provided superior bleeding control at Day 5 and survival benefit at Week 6, regardless of severity of liver failure or use of rescue TIPS. These findings highlight ESs as the optimal tamponade device, although they might not obviate the need for prompt rescue TIPS placement.

https://doi.org/10.1016/j.jhepr.2025.101581
© 2025 The Authors. Published by Elsevier B.V. on behalf of European Association for the Study of the Liver (EASL). This is an open access article under the CC BY license (http://creativecommons.org/licenses/by/4.0/).

Improved survival in patients with refractory variceal bleeding treated with esophageal stents: A multicenter cohort study

Delphine Weil[1,2,*], Morgane Clément[1], Charlotte Bouzbib[3], Jean-Paul Cervoni[1], Andrimalala Raoto[4], Grégoire Boivineau[5], Isabelle Ollivier-Hourmand[6], Noémi Reboux[7], Caroline Lemaitre[8], Cassandra Rayer[9], Marine Camus-Duboc[10], Ludovic Caillo[11], André-Jean Remy[12], Laure Elkrief[13], Guillaume Conroy[14], Faustine Wartel[15], Armand Garioud[16], Maeva Guillaume[17], Edouard Bardou-Jacquet[9,18], Stéphane Koch[19], Jean-Pierre Arpurt[4], Marika Rudler[3], Vincent Di Martino[1,2], ANGH, CREGG, SFED, GRAPHE, CFHTP groups

JHEP Reports **2025**. vol. 7 | 1–10

Check for updates

Background & Aims: Tamponade is a bridge therapy for refractory variceal bleeding. This study compared esophageal stents (ESs) and balloon tamponade (BT) in terms of early bleeding control and mortality.

Methods: We analyzed a cohort of patients with cirrhosis treated with tamponade in nine French hospitals between 2002 and 2023. The primary outcome was 6-week mortality. Multivariable analyses included Cox with time-dependent covariates and logistic regression models, adjusted for model for end-stage liver disease (MELD) score, rescue transjugular intrahepatic portosystemic shunt (rTIPS), and inverse probability of treatment weighting (IPTW) for ES.

Results: Sixty-three patients (87.3% male; mean age 55 years; 73.0% Child-Pugh C) were included. ES was used in 30 patients, BT in 33, and rTIPS subsequently in 20. Endoscopic control was attempted in 66.1% of cases. Adverse events were more frequent with ES (56.7% *vs.* 27.3%, p = 0.018), mostly stent migrations without clinical consequence, but less severe than with BT (two esophageal ruptures). Mortality was 42.9% (n = 27) at Day 5 and 55.6% (n = 35) at 6 weeks. Univariable analysis revealed lower mortality with ES than with BT (20.0% *vs.* 63.6% on Day 5, p = 0.0005 and 36.7% *vs.* 72.7% on Week 6, p = 0.0015) and with rTIPS (10.0% *vs.* 58.1% on Day 5, p = 0.0003 and 25.5% *vs.* 67.5% on Week 6, p = 0.0009). IPTW-adjusted and IPTW-weighted time-dependent Cox and logistic models confirmed decreased 6-week mortality (hazard ratio (HR) = 0.26, p = 0.0078) and better 5-day bleeding control (odds ratio (OR) = 6.1, p = 0.020) with ES.

Conclusions: In patients with cirrhosis-related refractory variceal bleeding, ESs were associated with significantly better early bleeding control and reduced mortality at both Day 5 and Week 6 compared with BT. Prospective studies are warranted to confirm these findings.

© 2025 The Authors. Published by Elsevier B.V. on behalf of European Association for the Study of the Liver (EASL). This is an open access article under the CC BY license (http://creativecommons.org/licenses/by/4.0/).

Introduction

Tamponade devices are used as a temporary measure in the event of refractory variceal bleeding, either massive at the outset or recurrent within the first 5 days, pending the availability of a more efficacious treatment of bleeding and/or portal hypertension.[1,2] Currently, two types of device are available: single (Linton) or double (Blakemore) balloon probes[3–5] and, more recently, expansive esophageal stents (ESs).[6–9]

In its most recent report, the Baveno Consortium suggested that the use of ESs was the optimal option, although it acknowledged that there were insufficient studies comparing the two categories of tamponade device.[10] Indeed, the literature reports only one randomized trial that included a very small number of patients, which was unable to demonstrate the superiority of either device in terms of immediate bleeding control or short- or mid-term survival.[11] The main arguments in favor of the use of ESs over balloons are their ease of placement, longer maintenance period, favorable safety profile, and the absence of esophageal lumen obstruction.[2,12]

Therefore, we conducted a comparative study to assess whether one device (balloon tamponade [BT] *vs.* ES) is superior to another in terms of short- and mid-term survival and bleeding control.

Patients and methods

Study design

This was a French multicenter cohort study that considered for inclusion patients from six general hospitals and nine university hospitals. A questionnaire was made available online to all French hepato-gastroenterologists from March 2021 to June 2023 by five French scientific societies involved in portal hypertension and digestive endoscopy, which promoted this study. Inclusions were both retrospective and prospective to

* Corresponding author. Address: Service d'Hépatologie et de Soins Intensifs Digestifs, CHU Jean Minjoz, 3 Bld Fleming, 25000 Besançon, France. Tel.: +33 381668421; fax: +33 381668417.
E-mail address: dweil@chu-besancon.fr (D. Weil).
https://doi.org/10.1016/j.jhepr.2025.101581

ensure a sufficient number of patients and to enable comparison of tamponade devices, covering a period from January 2002 to April 2023.

Ethics statement

The research was conducted in accordance with the Declarations of Helsinki and Istanbul, and the French Regulatory Authority for clinical studies. The Institutional Board 'Clinical Research and Innovation Department' and the Human Protection Committee East Area II Besancon, France, approved this study. A certificate from the President of the Human Protection Committee East Area II Besancon, France indicates that, according to the French Regulatory Authority for Clinical Studies, prospective and retrospective observational studies are not evaluated by Human Protection Committees. No informed consent was required for this study based on French Regulatory Authority guidance.

Outcome measures

Mortality at Week 6 was the primary endpoint. The secondary endpoints were 5-day mortality, 5-day bleeding control, bleeding recurrence, and tamponade device-related adverse events (AEs). Failure to control the initial bleeding is defined as massive bleeding or early rebleeding within the first 5 days after the index bleeding.[10] Therefore, we defined recurrent bleeding as occurring after Day 5 and collected these data on Day 7 and Week 6. Device-related AEs were recorded during insertion and after removal. Owing to the variable length of device maintenance, data were collected on Day 7.

Population

All patients who underwent tamponade for variceal bleeding between March 2021 and April 2023 and met the inclusion criteria were prospectively included in the study. The inclusion criteria were as follows: age >18 years; esophageal variceal bleeding requiring hospitalization and refractory to conventional treatment, and use of tamponade with a balloon catheter or covered self-expanding ES. Patients included in a center reporting the use of a single tamponade device were excluded from the study. Additional exclusion criteria included the existence of gastric varices, non-cirrhotic portal hypertension, or hepatocellular carcinoma. To increase the number of patients, investigators were encouraged to retrospectively include additional consecutive patients who had been hospitalized at their center between 2002 and 2021 based on data from the French National Uniform Hospital Discharge Data Set Database (PMSI) (International Classification of Diseases (ICD)-10 code: EHBD001). However, the timeframe used for the retrospective inclusion was based on the judgment of each investigator. The diagnosis of cirrhosis was based on conventional clinical, biochemical, radiological, or histological criteria. The severity of cirrhosis at the time of bleeding was assessed using the Child-Pugh and model for end-stage liver disease (MELD) scores.

Data collection

The questionnaire included 177 variables, of which 101 were collected on admission, 49 on Day 7, and 27 at Week six. Three variables were demographic, 18 were related to history of portal hypertension, 53 to characteristics and management of bleeding before tamponade, 23 to tamponade modalities, and 80 to outcome after tamponade. Of the post-tamponade follow-up data, 10 variables were related to AEs associated with the tamponade device, four to bleeding recurrence, and six to mortality. In the event of death, the investigator reported the cause. The use of rescue transjugular intrahepatic portosystemic shunt (rTIPS), defined as TIPS placement within the first 5 days to control bleeding, was also recorded and included four variables.

Statistical analysis

Results were compared between the BT and ES groups. Quantitative variables were expressed as mean ± SD when normally distributed, and qualitative variables as absolute numbers and percentages. Univariable comparisons were performed using the Student's *t* test or Mann-Whitney *U* test for quantitative variables, and the Chi-square or Fisher's exact test for qualitative variables, as appropriate. Survival analyses were conducted using the Kaplan-Meier method, with group comparisons assessed by the Log-rank test.

To adjust for baseline differences and minimize confounding factors, we used multivariable Cox and logistic regression models with inverse probability of treatment weighting (IPTW), derived from a selected propensity score (PS) model (PS3; Materials and methods S1A, S1B, S1C, Table S1, Table S2). The primary analyses were based on a stabilized and truncated IPTW (stIPTW3), applied directly to the models to improve covariate balance while limiting variance inflation. In addition, stIPTW3 was also incorporated as a covariate in separate sensitivity models to explore the consistency of results across alternative adjustment strategies. A time-dependent Cox model was used to account for the immortal time bias introduced by rTIPS placement when analyzing 6-week survival. Variables included in the multivariable models were chosen based on clinical relevance, univariable associations, and the absence of collinearity, adhering to the 10 events per variable rule.

In addition to models using different adjustment strategies (unadjusted, PS-adjusted, and alternative IPTW specifications; Tables S2 and S6), sensitivity analyses were conducted in two contexts. First, the analysis was restricted to patients included during the period when ESs were available (December 2010 to April 2023) to ensure treatment equipoise across centers (Fig. S2 and Table S5A). Second, to address immortal time bias related to delayed transjugular intrahepatic portosystemic shunt (TIPS) placement, a landmark analysis was performed that excluded all patients who died before Day 2 (Fig. S3 and Table S5B). All analyses were performed using NCSS 2019 (Kaysville, UT, USA) and R software, version 4.5.0.

Results

Study population

Characteristics

The study included 63 patients (33 in the BT group and 30 in the ES group) who were selected from 99 patients recorded from 15 centers (Fig. 1). All of the included patients came from the nine centers that reported using both ES and BT for tamponade (Fig. S1 shows inclusion timeline by center and device availability). The patients had cirrhosis-related portal

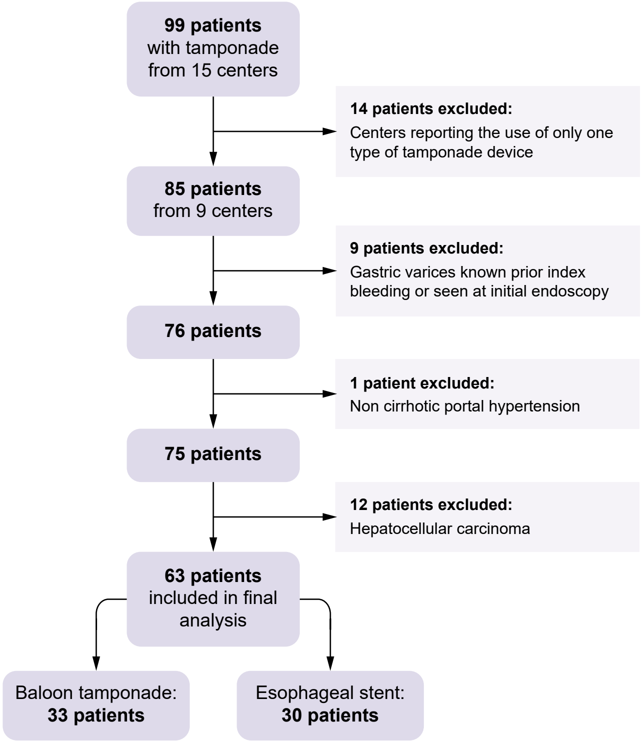

Fig. 1. Study flow chart. Flow chart showing the selection process for inclusion in the nationwide multicenter cohort. Among 99 patients with tamponade for variceal bleeding identified across 15 centers, 63 patients fulfilled the eligibility criteria and were included in the analysis. Reasons for exclusion are detailed, including patients from centers reporting the use of only one type of tamponade device, non-cirrhotic portal hypertension, gastric varices, and hepatocellular carcinoma.

hypertension and no gastric varices or hepatocellular carcinoma (Fig. 1). The baseline characteristics of the study population are shown in Table 1. Three centers were able to use ES before March, 2021 (in 11 patients). The other centers used ES after they became eligible for reimbursement in 2021. Of the 63 patients, 31 were enrolled prospectively (19 ES *vs.* 12 BT) and 32 retrospectively (11 ES *vs.* 21 BT). The proportion of patients receiving ES was higher among those enrolled prospectively *vs.* those enrolled retrospectively (61.3% *vs.* 38.7%, respectively; p = 0.032). Most patients were men (87.3%), with a mean age of 55 ± 13 years. Cirrhosis was alcohol related in 57 patients (90.5%). Most patients (73.0%) had Child-Pugh class C cirrhosis. The mean MELD score was 22.1 ± 9.0. Esophageal varices were documented in 61.9% of patients before the index bleeding episode. Thirty-one patients (49.2%) had previously experienced at least one variceal bleeding event. Of these patients, 15 met the criteria for pre-emptive (p)TIPS[13] at the time of previous bleeding, and five received TIPS. Of these five patients, three had clinical ascites despite TIPS and one had TIPS thrombosis. The median time between the two bleeding episodes was 13 days for patients with pTIPS criteria during the first episode *vs.* 133 days for other patients. No patient received anticoagulant treatment, whereas only two patients in each group received antiplatelet agents. Baseline hemodynamic parameters were comparable between the two groups. A mean arterial pressure (MAP) <60 mmHg was observed in 31.6% of cases. Mean hemoglobin concentration was 7.1 ± 2.2 g/dl, and mean creatinine was 113 ± 88 µmol/L.

The baseline characteristics of patients in both groups served as the basis for constructing multiple PS models to predict the probability of receiving ES *vs.* BT and to select the model that best balanced covariates between groups (Materials and methods S1). The effectiveness of the final weighting strategy, based on stIPTW3, is illustrated in Fig. 2.

First-line management of the bleeding episode

Management before tamponade is summarized in Table 2. More than half of the patients (52.4%) were admitted directly to

Table 1. Baseline characteristics.

Variable	BT (n = 33)	ES (n = 30)	Total (n = 63)	*p* value
Male sex	28 (84.8%)	27 (90.0%)	55 (87.3%)	0.540
Age (years)	55.1 ± 12.5	55.3 ± 12.8	55.2 ± 12.6	0.940
Prospective enrollment	12 (36.4%)	19 (63.3%)	31 (49.2%)	0.032
Alcohol-related cirrhosis	27 (90.9%)	30 (90.0%)	57 (90.5%)	0.902
MELD score	20.6 ± 8.9	23.7 ± 8.9	22.1 ± 9.0	0.172
Child-Pugh A stage	2 (6.1%)	0 (0.0%)	2 (3.2%)	0.270
Child-Pugh B stage	5 (15.1%)	10 (33.3%)	15 (23.8%)	0.091
Child -Pugh C stage	26 (78.8%)	20 (66.7%)	46 (73.0%)	0.279
Known esophageal varices	19 (57.6%)	20 (66.7%)	39 (61.9%)	0.458
Known previous decompensation	19 (57.6%)	24 (80.0%)	43 (68.2%)	0.056
History of variceal bleeding	15 (45.4%)	16 (53.3%)	31 (49.2%)	0.532
Previous TIPS	1 (3.0%)	4 (13.3%)	5 (7.9%)	0.131
Non-selective beta blockers	8 (24.2%)	10 (33.3%)	18 (28.6%)	0.425
Diuretics	7 (21.2%)	10 (33.3%)	17 (27.0%)	0.279
Antiplatelet agents	2 (6.1%)	2 (6.7%)	4 (6.3%)	0.921
Ascites	18/32 (56.2%)	19 (63.3%)	37/62 (56.4%)	0.569
Hepatic encephalopathy	18/32 (56.2%)	17 (56.7%)	35/62 (51.0%)	0.535
Mean arterial pressure (mmHg)	64.1 ± 21.8	65.7 ± 24.6	68.4 ± 18.0	0.790
Hemoglobin (g/dl)	7.7 ± 2.4	6.6 ± 1.9	7.1 ± 2.2	0.051
Prothrombin time (%)	38.9 ± 18.0	33.6 ± 16.3	36.3 ± 17.3	0.222
Bilirubin (µmol/L)	92.6 ± 101.0	121.2 ± 170.1	106.7 ± 138.9	0.425
Albumin (g/L)	25.0 ± 6.8	24.7 ± 4.9	24.8 ± 5.9	0.834
Creatinine (µmol/L)	105.7 ± 73.1	121.2 ± 102.7	113.3 ± 88.5	0.499

Statistical differences were assessed using Chi-squared or Fisher's exact tests for percentages and Student t test for mean differences. BT, balloon tamponade; ES, esophageal stent; MELD, model for end-stage liver disease; TIPS, transjugular portosystemic shunt.

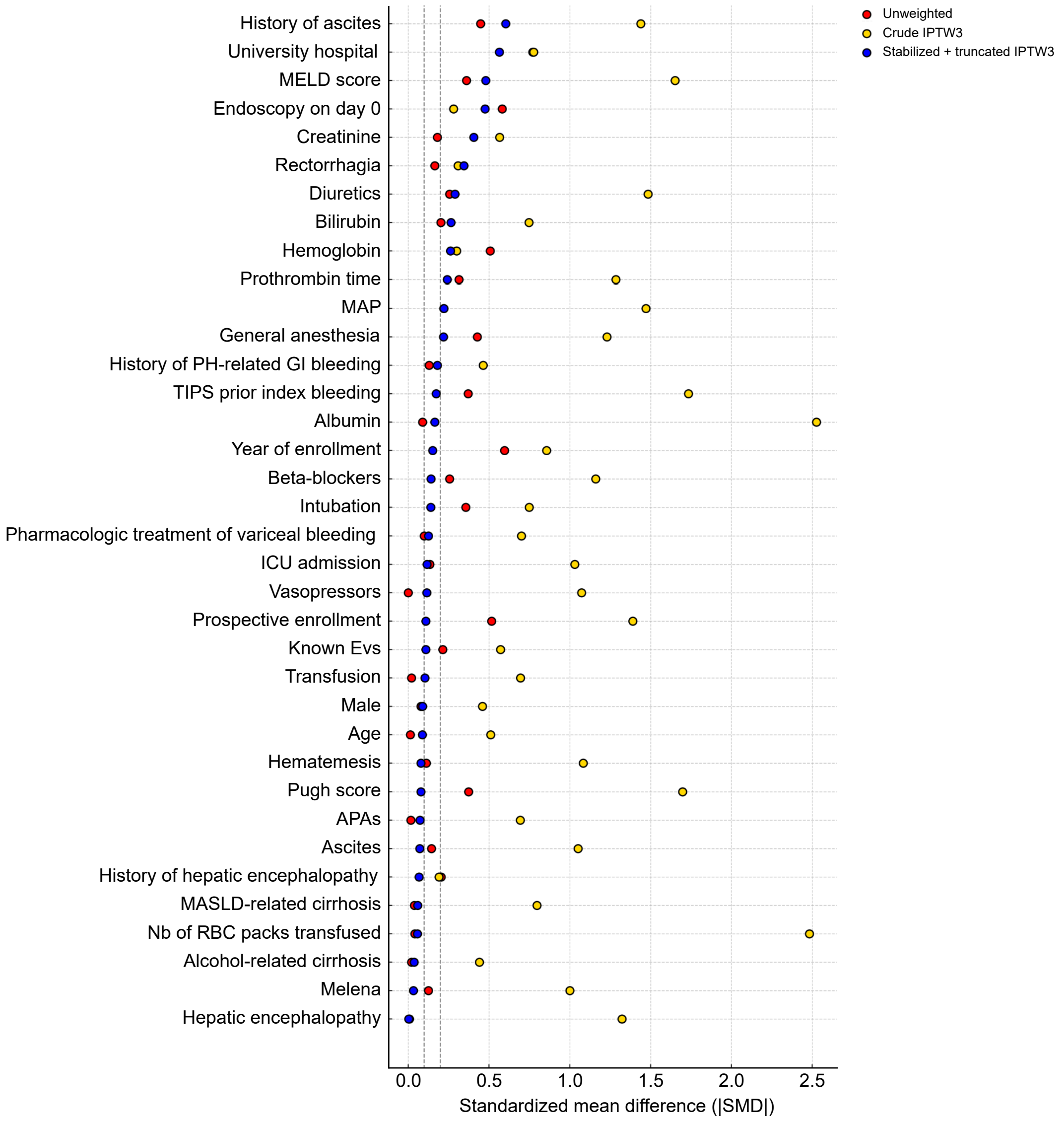

Fig. 2. Covariate balance before and after IPTW derived from PS model 3. Love plot showing SMDs for 36 baseline covariates before weighting (red), after crude IPTW using PS3 (yellow), and after stabilized/truncated IPTW using PS3 (blue). SMDs were calculated as mean or proportion differences divided by pooled standard deviation. While crude IPTW failed to correct baseline imbalances, the final weighting strategy substantially improved covariate balance (SMD <0.2 for 23/36 variables; only two variables had SMD >0.5), including key factors, such as year of inclusion and prospective enrollment. The choice of PS3 among 11 PS models is detailed in Table S1. Full balance metrics are provided in Table S2. APA, antiplatelet agent; EV, esophageal varices; GI, gastrointestinal; ICU, intensive care unit; IPTW, inverse probability of treatment weighting; MAP, mean arterial pressure; MASLD, metabolic dysfunction-associated steatotic liver disease; MELD, model for end-stage liver disease; PH, portal hypertension; PS, propensity score; RBC, red blood cell; SMD, standardized mean difference; TIPS, transjugular intrahepatic portosystemic shunt.

the intensive care unit (ICU). Most patients (90.5%) required blood transfusion, with a mean number of packed red cells of 5.0 units. Antibiotic prophylaxis was prescribed to 57 of 63 patients. On admission, 57 patients underwent endoscopy. This procedure was performed in sufficiently stable patients, and more frequently in the ES group than in the BT group (100.0% *vs.* 81.8%, p = 0.014). Endoscopic findings confirmed the presence of grade II (42.5%) or III (55.3%) esophageal varices. Endoscopy performed before tamponade confirmed the esophageal origin of bleeding in all patients. Endoscopic hemostasis was attempted in 70.4% of patients in the BT group compared with 62.1% of patients in the ES group (p = 0.512). The most common method of hemostasis was band ligation, used in 32 patients. Post-banding ulcers were diagnosed in 11 patients, with a higher prevalence in the ES group than in the BT group (31.0% *vs.* 7.4%, respectively; p = 0.026). The proportion of patients with persistent bleeding despite endoscopic and pharmacological treatment (rather than massive bleeding) did not significantly differ between the ES and BT groups (56.7% *vs.* 45.2%, respectively; p = 0.369). The interval between the onset of variceal bleeding and placement of the tamponade device was 14 ± 28 h in the ES group and 12 ± 22 h in the BT group (p = 0.661). The time required to place the tamponade device (data available from only 14 patients) was longer in the ES group than in the BT group (16 ± 7 min *vs.* 8 ± 3 min; p = 0.021). The technical success rate of tamponade was 93.9% in the BT group and 93.3% in the ES group (p = 0.922). Technical difficulties with

Table 2. Initial management of bleeding before tamponade.

Variables	BT	ES	Total	*p* value
Care in a university hospital	30 (90.9%)	17 (56.7%)	47 (74.6%)	0.002
Intensive Care Unit admission	16 (48.5%)	17 (56.7%)	33 (52.4%)	0.516
Blood transfusion	30 (90.9%)	27 (90.0%)	57 (90.5 %)	0.903
Number of red blood cell packs (mean±SD)	5.2 ± 4.5	4.7 ± 3.0	5.0 ± 3.8	0.613
Vasopressive amines	16 (48.5%)	15 (50.0%)	31 (49.2%)	0.904
Vasoactive drugs for portal hypertension	30 (90.9%)	28 (93.3 %)	58 (92.1%)	0.722
Antibiotic prophylaxis	28 (84.8%)	29 (96.7%)	57 (90.5%)	0.111
Intubation	17 (51.5%)	15 (50.0%)	32 (50.8%)	0.904
Endoscopy at day 0	27 (81.8%)	30 (100.0%)	57 (90.5%)	0.014
Esophageal varices stage III*	15 (55.5%)	16 (55.1%)	31 (55.3%)	0.977
Active bleeding*	25 (92.6%)	23 (79.3%)	48 (85.7%)	0.156
Adherent clot*	6 (22.2%)	4 (13.8%)	10 (17.9%)	0.410
Platelet clot*	3 (11.1%)	7 (24.1%)	10 (17.9%)	0.203
Post banding ulcer*	2 (7.4%)	9 (31.0%)	11 (19.6%)	0.026
Gastropathy*	2 (7.4%)	7 (24.1%)	9 (16.1%)	0.088
Endoscopic hemostasis attempt*	19 (70.4%)	18 (62.1%)	37 (66.1%)	0.512
Band ligation*	17 (63.0%)	15 (51.7 %)	32 (57.1%)	0.396
Glue obliteration*	0 (0.0%)	1 (3.4%)	1 (1.8%)	0.834
Sclerosis*	5 (18.5%)	1 (3.4%)	6 (10.7%)	0.068

Statistical differences were assessed using Chi-squared or Fisher's exact tests for percentages and Student t test for mean differences. BT, balloon tamponade; ES, esophageal stent; ICU, intensive care unit.
*Data reported for 56 patients (27 in BT group; 29 in ES group).

the self-expanding stent were reported in three cases: one probe was deemed too rigid to pass through the esophageal lumen, one migrated immediately during insertion, and another could not be released because of a lack of gastric balloon expansion. Two instances of failed balloon probe insertion were reported, including one case of esophageal rupture. Overall, two patients in whom the ES procedure failed received a Blakemore probe, and one patient in whom the BT procedure failed received an ES. Given that conversion was performed immediately, assignment to the ES or BT group was based on the most recent device placed. The tamponade device was maintained for an average of 1.5 ± 2.0 days in the BT group, compared with 6.7 ± 4.3 days in the ES group (p = 0.001).

Rescue TIPS placement

The decision to implant rTIPS was made at the discretion of the referring physician. None of the available baseline variables, including indicators of severity (*i.e.* high MELD score, ICU admission, intubation, renal replacement therapy, mean arterial pressure, use of vasopressor amines, and volume of blood transfusion at Day 0), could discriminate subsequent use of rTIPS. In addition, the date and site of enrollment did not affect access to rTIPS, which was implanted in patients from the nine participating centers. Within the first 5 days, 12 patients in the ES group and eight patients in the BT group underwent rTIPS placement (including two bare stents in the BT group). There was no significant difference in rate of use of rTIPS between groups (ES: 19.1% *vs.* BT: 12.7%; p = 0.180), period (prospective enrollment or not: 38.7% *vs.* 25.0%, respectively; p = 0.242) or time taken to place the rTIPS (1.3 ± 0.5 days in the BT group *vs.* 1.9 ± 1.4 days in the ES group; p = 0.339). No TIPS was placed between Day 5 and Week 6.

Mortality

The overall mortality rate on Day 5 was 42.9% (n = 27). Eight additional patients died between Day 5 and Week 6 (Table 3). Refractory hemorrhagic shock was the primary cause of mortality on Day 5 (79.2% of cases), whereas multiorgan failure accounted for 8.3% of deaths. The 6-week mortality rate was 55.6%. Recurrent bleeding was the cause of death in 37.5% of cases between Day 5 and Week 6, whereas sepsis was involved in 25.0% of cases and multiorgan failure in 37.5%.

Impact of the tamponade device

Six-week mortality was significantly lower in the ES group compared with the BT group (36.7% *vs.* 72.7%, respectively; p = 0.0015; Table 3 and Fig. 3; Figs S4 and S5), with a similar difference already present at Day 5 (20.0% *vs.* 63.6%, respectively; p = 0.0005). Early mortality within the first 2 days was also significantly lower in the ES group (13.3% *vs.* 51.2%, respectively, p = 0.0013). This survival difference remained significant in a sensitivity analysis restricted to the period during which ES was available (December 2010 to April 2023; Fig. S2). Time-dependent Cox models adjusted for MELD score, rTIPS, and stIPTW3 confirmed that ES was independently associated with improved 6-week survival (Table 4; Table S3). A landmark analysis restricted to patients alive on Day 2 yielded consistent results (Table S5B). When further restricted to patients alive on Day 5, the association was no longer statistically significant.

Impact of rescue TIPS

Among patients who survived for 6 weeks, 47.4% (9/19) in the ES group and 66.7% (6/9) in the BT group had undergone rTIPS. Regardless of the tamponade device used, the 6-week mortality was significantly lower in patients who underwent rTIPS (25.5% *vs.* 67.5% in patients who did not undergo rTIPS; p = 0.0009, Fig S3A, S5). This association is further illustrated in Fig S3B which confirms the protective effect of rTIPS on 6-week survival in a landmark sensitivity analysis restricted to patients who survived the first 2 days. The Day 5 mortality was also significantly lower in patients who underwent rTIPS than in those who did not (10.0% *vs.* 58.1%, respectively; p = 0.0003). In the ES group, Day 5 and Week 6 mortality rates were 8.3% and 25.0%, respectively, with rTIPS, and 27.8% and 44.4%, respectively, without rTIPS (Log-rank test; p = 0.248). In the BT

Table 3. Outcome events.

Variable	BT	ES	Total	*p* values
Mortality at Week 6	24 (72.7%)	11 (36.7%)	35 (55.6%)	0.0015
Mortality at Day 5	21 (63.6%)	6 (20.0%)	27 (42.9%)	0.0005
Control of bleeding at Day 5	10 (30.3%)	21 (70.0%)	31 (49.2%)	0.002
Recurrent bleeding at Day 7*	1/12 (8.3%)	2/23 (8.3%)	3/36 (8.3%)	1.000
Recurrent bleeding at Week 6*	1/10 (10.0%)	2/23 (8.7%)	3/33 (9.1%)	0.905
**Device-related AEs**				
At least one device-related AE	9 (27.3%)	17 (56.7%)	26 (41.3%)	0.018
Esophageal rupture	2 (6.1%)	0 (0.0%)	2 (3.2%)	0.493
Inhalation pneumonia	5 (15.2%)	3 (10.0%)	8 (12.7%)	0.539
Placement-related esophageal injury	4 (12.1%)	7 (23.3%)	11 (17.5%)	0.242
Chest pain	2 (6.1%)	2 (6.7 %)	4 (6.4%)	0.921
Device migration	0 (0.0%)	8 (26.7%)	8 (12.7%)	0.0015
Early withdrawal	2 (6.1%)	2 (6.7%)	4 (6.4%)	0.921
Bleeding after removal	0/7 (0.0%)	4/11 (36.4%)	4/18 (22.2%)	0.070
Ischemia after removal	0/11 (0.0%)	3/22 (13.7%)	3/33 (9.1%)	0.199
**Other OEs**				
Hepatic encephalopathy	10 (30.3%)	17 (56.7%)	27 (42.9%)	0.035
Infection	8 (24.2%)	9 (30.0%)	17 (27.0%)	0.607
Hepatorenal syndrome	1 (3.3%)	7 (23.3%)	8 (13.3%)	0.023
Cardiac arrest	4 (12.1%)	0 (0.0%)	4 (6.4%)	0.115

Figures are given for 33 patients in the BT group and 30 in the ES group. If data are missing for some variables, the denominators are given. Statistical differences were assessed using Chi-squared or Fisher's exact test.
AE, adverse event; BT, balloon tamponade; ES, esophageal stent; OE, outcome event.
*Analysis excludes patients with uncontrolled bleeding on Day 5.

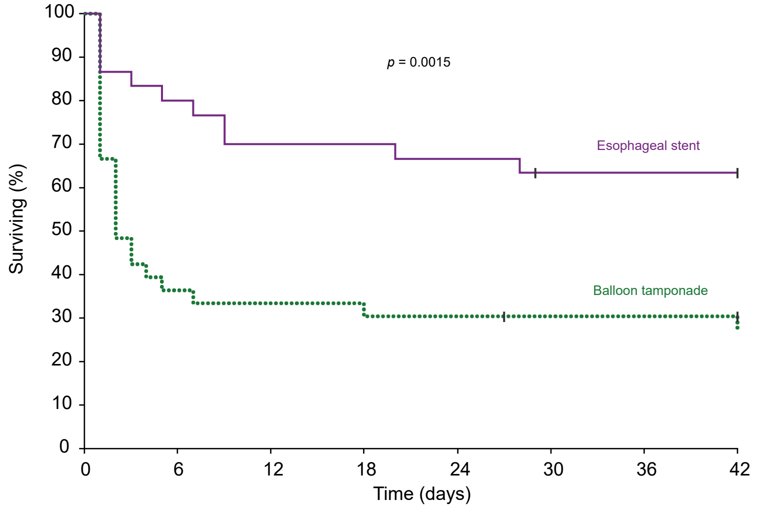

No. at risk								
Balloon tamponade	33	12	11	11	10	9	9	9
Esophageal stent	30	24	21	21	20	18	18	18

Fig. 3. Survival up to 6 weeks according to the type of tamponade device used. Unadjusted overall survival at Day 42 (Week 6) was significantly higher in patients treated with an ES compared with PT (Log-rank test, $p = 0.0015$). This survival analysis shows a significant survival benefit in favor of ESs, with early and sustained divergence of the curves, emphasizing the impact of Ess on early mortality reduction. BT, balloon tamponade; ES, esophageal stent.

group, the mortality rates on Day 5 and Week 6 were 12.5% and 25.0%, respectively, with rTIPS and 80.0% and 84.0%, respectively, without rTIPS (Log-rank test; $p = 0.0013$).

Multivariable analysis

In the stIPTW3-adjusted time-dependent multivariable Cox model on mortality at Week 6, high MELD scores were associated with a significantly increased risk of death (hazard ratio [HR] = 1.056, 95% CI 1.018–1.094, $p = 0.0035$), whereas the use of ES (HR = 0.261; 95% CI 0.097–0.702, $p = 0.0078$) and rTIPS (HR = 0.211; 95% CI 0.059–0.752, $p = 0.0165$) were both found to be independent protective factors (Table S3). The robustness of these findings was tested in multivariable models weighted by stIPTW3 or incorporating stIPTW3 as a covariate, and multiple sensitivity analyses with alternative adjustment strategies (unadjusted, PS3-adjusted, adjusted with crude IPTW3, and stIPTW3 Cox models with no time-dependent covariates) (Table 4; Table S6). All models consistently supported the beneficial effects of ES on survival (Table 4).

Table 4. Impact of ES on Week-6 mortality and early bleeding control across univariable and multivariable models.

Analysis	HR	95% CI	*p* values
**Impact of esophageal stent on Week-6 mortality**			
Univariable Cox regression	0.328	0.159–0.674	0.0024
Multivariable time-dependent Cox (ES + MELD* + rTIPS†) without PS-derived adjustment	0.229	0.105–0.498	0.0002
Multivariable time-dependent Cox (ES + MELD* + rTIPS‡ + stIPTW3 as covariate)	0.261	0.097–0.702	0.0078
Multivariable time-dependent Cox (ES+ MELD* + rTIPS‡) weighted by stIPTW3	0.269	0.119–0.605	0.0015
**Impact of ES on early bleeding control**			
Univariable analysis (Chi-squared)	5.367	1.827–15.763	0.0016
Multivariable logistic regression (ES + MELD* + rTIPS‡) without PS-derived adjustment	8.999	2.623–30.873	0.0005
Multivariable logistic regression (ES + MELD* + rTIPS‡ + stIPTW3* as covariate)	6.127	1.323–28.376	0.0205
Multivariable logistic regression (ES + MELD* + rTIPS‡) weighted by stIPTW3	14.716	3.145–68.865	0.0006

The use of ESs was consistently associated with improved outcomes in patients with refractory variceal bleeding. Across all models, their use resulted in a reduction in Week-6 mortality of at least 65%, and an ~five fold increase in the likelihood of achieving early bleeding control compared with BT. The magnitude and consistency of these effects across univariable and multivariable models underscore the robustness of this therapeutic benefit.

BT, ballon tamponade; CI, confidence interval; ES, esophageal stent; HR, hazard ratio; IPTW, inverse probability of treatment (ES) weighting; MELD, model for end stage liver disease; OR, odds ratio; TIPS, transjugular portosystemic shunt.

*Expressed as continuous variables.

†rTIPS was included as a time-dependent variable in the Cox model.

Bleeding

Control of initial bleeding

Control of initial bleeding was observed in 31 patients (49.2%), more frequently in the ES group than in the BT group (70.0% *vs.* 30.3%, respectively; $p = 0.002$; Table 3). Control of initial bleeding was also more frequent in the 20 patients who underwent rTIPS placement compared with those who did not (70.0% *vs.* 39.5%, respectively; $p = 0.024$). In patients who underwent rTIPS, control of initial bleeding did not differ between the ES and BT groups (75.0% *vs.* 62.5%, respectively; $p = 0.550$). Conversely, in the 43 patients who did not undergo rTIPS, bleeding control was observed more frequently in the ES group than in the BT group (66.7% *vs.* 20.0%, respectively; $p = 0.002$).

In the stIPTW3-adjusted logistic regression analysis, low MELD score (odds ratio [OR] = 1.088, 95% CI 1.031–1.148, $p = 0.040$), rTIPS (OR = 3.955, 95% CI 1.065–14.689, $p = 0.0145$), and ES (OR = 6.127, 95% CI 1.323–28.376, $p = 0.0205$) were independently associated with bleeding control at Day 5 (Table S4). Similar conclusions were reached in the stIPTW3-weighted logistic regression analysis, except for rTIPS, which had no independent effect (Table S4). Multivariable models that explored different adjustment strategies reinforced the robustness of the observed benefits of ES in achieving early bleeding control (Table 4, Table S6).

Recurrent bleeding

Of the 36 patients who survived to Day five (11 in the ES group with rTIPS, 13 in the ES group without rTIPS, seven in the BT group with rTIPS, and five in the BT group without rTIPS), two patients experienced recurrent bleeding on Day 7 in the ES group and one in the BT group. Two of these three had received rTIPS (one after ES, one after BT). Three additional episodes of recurrent bleeding were observed at Week 6. These episodes occurred in two patients in the ES group and one in the BT group. No significant differences were observed for recurrent bleeding between the groups.

Other outcome events

Table 3 presents the outcomes observed throughout the follow-up period. No patient underwent liver transplantation within the first 6 weeks following the index variceal bleeding. With regard to device-related AEs, the available records indicate that four cases of esophageal bleeding and three cases of ischemia on stent removal were reported in the ES group. By contrast, no such complications occurred in the BT group. Two cases of esophageal rupture and eight cases of stent migration were observed in the BT and ES groups, respectively. The occurrence of esophageal ulcers, inhalation pneumonia, chest pain, and early withdrawal did not differ between the two groups. Overall, 17 patients in the ES group and nine patients in the BT group (56.7% *vs.* 27.3%, respectively; $p = 0.018$) experienced at least one device-related AE. Most of these events involved stent migration (Table 3) without clinical consequences. The occurrence of hepatic encephalopathy or hepatorenal syndrome at Week 6 was more frequent in the ES group than in the BT group (Table 3), but the differences were not significant when considering only patients alive at Day 5.

Discussion

Refractory variceal bleeding, defined as bleeding that persists despite pharmacological and endoscopic treatments, is a rare but serious complication of cirrhosis. It accounts for an estimated 15–20% of variceal bleeding cases.[14,15] Despite the use of rTIPS, mortality rates in different studies range from 20% to 50%.[5,12] In this context, any contribution to improving survival would be welcome.

In this nationwide multicenter cohort study of patients with refractory variceal bleeding, self-expanding ESs were associated with significantly improved early bleeding control and reduced mortality on Day 5 and Week 6, compared with BT. To the best of our knowledge, this is the first comparative study to document the superiority of ES in this setting. These associations remained consistent following rigorous adjustment using stIPTW3, based on a well-performing PS model that included 23 pretreatment covariates. Notably, the 5-day mortality rate decreased from 63.6% with BT to 20.0% with ES, and the 6-week mortality rate decreased from 72.7% to 36.7%. This dramatic reduction in early mortality further underscores the protective role of ESs during the acute phase of bleeding (Fig. 3). The highest survival rates were observed in patients who received both ES and rTIPS, with 91.7% of patients alive at Day 5 and 75% alive at Week 6. The benefit of ES

observed was not only statistically robust, but also clinically meaningful, especially considering the severity of illness in this cohort (MELD 22.1 and 73.0% Child-Pugh C). The impact on bleeding control persisted as a significant factor in fully adjusted models (OR = 6.127, p = 0.0205; Table 4; Table S4). In addition, the ES was found to be independently associated with decreased 6-week mortality (HR = 0.261, p = 0.0078; Table 4; Table S3). The survival benefit resulting from ES was noteworthy and somewhat unexpected in its magnitude. Despite the frequent use of rTIPS during the study period, which did not differ between groups, and the significant impact of liver failure, an unlikely influence of a tamponade device,[16] the survival benefit associated with ES remained unaffected. rTIPS likewise provided protection in most cases, although its statistical significance varied depending on the modeling strategy. Taken together, these findings support a sequential strategy in which tamponade ensures immediate mechanical compression to stop bleeding, and rTIPS offers definitive treatment for portal hypertension. While the best outcomes were seen with a combination of ES and rTIPS, ES alone was also associated with improved survival.

The credibility of these results is bolstered by several factors. The cohort included patients from nine centers, encompassing the main configurations of care delivery, including university and non-university hospitals, with or without ICUs, and with or without local TIPS availability. Although it was not possible to formally confirm strict consecutive inclusion because of potential limitations in ICD-based retrospective identification, the pragmatic design and efforts made to include as many patients as possible through national hepatology networks suggest good representativeness. Thirty-one patients were prospectively enrolled in the study, and the remainder were included retrospectively based on consistent diagnostic coding. The duration of inclusion varied across centers. However, a centralized methodology and standardized data collection processes minimized heterogeneity. Two additional methodological strengths should be noted. First, the study was restricted to centers that had used both tamponade devices during the inclusion period, which limited the risk of center-level confounding. Second, rigorous eligibility criteria were implemented to ensure a homogeneous population (Fig. 1). Only patients with cirrhosis were included, and all patients with hepatocellular carcinoma were excluded because it can compromise the optimal management of variceal bleeding, particularly access to ICUs. Any patient with gastric varices, even if presumed non-bleeding, was also excluded to avoid introducing confusion in the analysis of esophageal compression efficacy on survival outcomes. The third PS model exhibited remarkable discrimination, as evidenced by an AUROC of 0.935 (Materials and methods S1B). In addition, substantial covariate balance was attained following the implementation of a weighting scheme. Sensitivity analyses using various adjustment methods (PS covariate, crude IPTW, and stIPTW3) consistently reproduced the effects of ES on both endpoints (Table S6).

However, the present study had several limitations. The study was observational in nature, and unmeasured confounding cannot be fully excluded despite the robust methodology used. The impact of rTIPS on survival appears to be less significant than that of ES, with varying levels of significance across multivariable models (Tables S3 and S4). Among the time-dependent Cox models, only the model that included stIPTW3 as a covariate showed a statistically significant protective effect of rTIPS. By contrast, the model weighted by stIPTW3 did not (Table S3). This discrepancy is likely the result of the temporal overlap between rTIPS timing and early mortality. Notably, 42.4% of deaths occurred within the first 2 days, during which >90% of rTIPS procedures were performed. Consequently, patients who died early were inherently excluded from the rTIPS group, thereby introducing immortal time bias in non-time-dependent analyses. Although time-dependent modeling addresses immortal time bias, it can yield unstable estimates in this context because of the high concentration of events within a narrow time window. This makes the analysis highly sensitive to minor imbalances between groups. Notably, all non-corrected models including unadjusted, IPTW-adjusted, and landmark analyses excluding early deaths, consistently demonstrated a survival benefit of rTIPS. The variability observed in the effect of rTIPS, as revealed by the analyses, is hypothesized to be driven primarily by analytic constraints rather than by true clinical inefficacy. Indeed, the protective effect of rTIPS was confirmed by a sensitivity analysis that excluded all patients who died before Day 2.

It is noteworthy that early deaths before Day 2 were considerably more prevalent in the BT group (51.2% *vs.* 13.3% in the ES group; p = 0.0013). However, the survival benefit associated with ES remained significant in the landmark analysis restricted to patients who survived at least 48 h (Table S5B). This finding reinforces the robustness of the ES effect and suggests that its protective association cannot be explained solely by a lead-time advantage or an imbalance in early deaths. Conversely, in analyses restricted to the prospectively enrolled subset, the beneficial effect of ES was no longer statistically significant, most likely because of insufficient power rather than an absence of effect.

In addition, there are certain contextual and methodological aspects that merit attention. First, access to ESs was initiated in December 2010 in a limited number of centers and subsequently became universal after 2021. This raises the possibility of a period effect (Fig. S1). However, the stIPTW3 model effectively neutralized differences in the year of inclusion and prospective enrollment between groups. This is evident from the standardized mean difference of <0.2 for both covariates (Fig. 2; Table S2. Furthermore, a sensitivity analysis restricted to the broader period from December 2010 to April 2023, corresponding to the period of potential ES availability, confirmed the observed benefit (Fig. S2 and Table S5A), thereby mitigating this concern. Second, the determination of causes of death was not centrally adjudicated, and decisions about rTIPS placement were not based on predefined criteria. This might indicate center-specific practices or implicit assessments of futility in critically ill patients,[17] which could introduce selection bias. Third, a randomized controlled trial would be the most robust design for validating these findings; however, such an approach is unlikely to be feasible. Refractory variceal bleeding requiring tamponade is a rare occurrence, and the French ICD records estimated that only 1.4% of variceal bleeding cases per year involved tamponade.[18] Furthermore, recent data from French and international registries indicate that tamponade was

used in only 103 (3.4%) of 3,019 patients enrolled over 5 years in 87 European centers.[19] The low incidence of the condition in question poses significant logistical and ethical challenges to implementing of large-scale randomized trials within a reasonable timeframe.

Another consideration is that ESs might create more favorable conditions for rTIPS placement by allowing for a longer tamponade duration and maintaining luminal patency. This was not directly measurable in the present study. This hypothesis is supported by the finding that an ES can remain in place for several days, ensuring hemodynamic stabilization and facilitating transfer to a tertiary care facility. Although the number of transfers was limited in this cohort, this effect might be underestimated because of the retrospective nature of some of the data. Consequently, the function of the ES might extend beyond achieving hemostasis immediately, thereby contributing to the success of subsequent therapeutic interventions.

In summary, the use of tamponade with a self-expanding stent should not be considered merely an interim measure while awaiting definitive therapy. Rather, it should be recognized as a crucial component of initial management. The substantial decrease in early mortality, especially when ES is used with timely rTIPS, supports a two-step therapeutic approach that could transform outcomes in this high-risk population. These findings should inform updates to international practice guidelines, including the Baveno Consensus, by recognizing the therapeutic value of ESs as integral interventions, rather than only bridges. Further prospective data are necessary to validate these findings and to optimize care pathways. This includes facilitating prompt access to specialized centers for TIPS placement.

Affiliations

[1]*Service d'Hépatologie et Soins Intensifs Digestifs, CHU Jean Minjoz, Besançon, France;* [2]*Université Marie et Louis Pasteur, EFS, INSERM UMR1098 RIGHT, Besançon, F-25000, France;* [3]*Unité de Soins Intensifs d'Hépatologie et Gastro-Entérologie, Hôpital Pitié-Salpêtrière, APHP, Paris, France;* [4]*Service d'Hépato-Gastroentérologie, CH d'Avignon, Avignon, France;* [5]*Service de Gastroentérologie, Hôpital Nord, AP-HM, Hôpital Nord AP-HM, Marseille, France;* [6]*Service d'Hépatologie, CHU Côte de Nacre, Caen, France;* [7]*Service d'Hépato-Gastroentérologie, CHU Cavale Blanche, Brest, France;* [8]*Service d'Hépato-Gastroentérologie, Groupe Hospitalier du Havre, Le Havre, France;* [9]*Service des Maladies du Foie, CHU de Rennes, France;* [10]*Centre d'Endoscopie Digestive, DMU SAPERE, Sorbonne Université, Hôpital Saint-Antoine AP-HP, Paris, France;* [11]*Service d'Hépato-Gastroentérologie, CHU de Nîmes, Université de Montpellier-Nimes, Nîmes, France;* [12]*Service d'Hépato-Gastroentérologie et Maladies de la Nutrition, CH de Perpignan, Perpignan, France;* [13]*Service d'Hépato-Gastroentérologie, Hôpital Trousseau - CHU de Tours, Tours, France;* [14]*Service d'Hépato-Gastroentérologie, CHR de Metz-Thionville Hôpital Mercy, Metz, France;* [15]*Service des Maladies de l'Appareil digestif et de la Nutrition, CH de Valenciennes, Valenciennes, France;* [16]*Service d'Hépato-Gastroentérologie, CHI Lucie et Raymond Aubrac, Villeneuve-Saint-Georges, France;* [17]*Service de Gastro-Entérologie, Clinique Pasteur, Toulouse, France;* [18]*Université de Rennes, INSERM Institut Numecan, Rennes, France;* [19]*Service de Gastroentérologie et Nutrition, Endoscopie Digestive, CHU Jean Minjoz, Besançon, France*

Abbreviations

AE, adverse event; APA, antiplatelet agent; BT, balloon tamponade; ES, esophageal stent; EV, esophageal varices; GI, gastrointestinal; HR, hazard ratio; ICD, International Classification of Diseases; ICU, intensive care unit; IPTW, inverse probability of treatment weighting; MAP, mean arterial pressure; MASLD, metabolic dysfunction-associated steatotic liver disease; MELD, model for end-stage liver disease; OE, outcome event; OR, odds ratio; PH, portal hypertension; PS, propensity score; pTIPS, pre-emptive transjugular intrahepatic portosystemic shunt; RBC, red blood cell; rTIPS, rescue transjugular intrahepatic portosystemic shunt; SMD, standardized mean difference; stIPTW3, stabilized and truncated IPTW; TIPS, transjugular intrahepatic portosystemic shunt.

Financial support

No funding was received for this study.

Conflicts of interest

The authors have no conflicts of interest to report.

Please refer to the accompanying ICMJE disclosure forms for further details.

Authors' contributions

Study conceptualization: DW, J-PC, SK, J-PA, MC-D, MG, MR. Methodology and questionnaire: DW, J-PC, MC, MR. Investigation and data collection: MC, CB, AR, GB, IO-H, NR, CL, CR, MC-D, LC, A-JR, LE, GC, FW, AG, EB-J, J-PA. Data curation and statistical analysis: VDM, DW. Drafting of manuscript: VDM, DW. Review and editing: DW, MC, CB, J-PC, AR, GB, IO-H, NR, CL, CR, MC-D, LC, A-JR, LE, GC, FW, AG, MG, EB-J, SK, J-PA, MR, and VDM. All authors approved the final version of the manuscript. Guarantor of manuscript: DW.

Data availability

Data collected for the study, including individual participant data, will be made available under transfer agreement from the corresponding author upon reasonable request. A signed data access agreement is required. The data provided will de-identify the participant data. There are restrictions on how the data can be used or how long the data will be available.

Acknowledgements

We thank Fiona Ecarnot for her editorial assistance. This study was presented at the French Liver Congress (AFEF) in October 2023 and at the AASLD annual meeting in November 2023.

Supplementary data

Supplementary data to this article can be found online at https://doi.org/10.1016/j.jhepr.2025.101581.

References

[1] Gralnek IM, Garcia-Pagan JC, Gea VH. Challenges in the management of esophagogastric varices and variceal hemorrhage in cirrhosis - a narrative review. Am J Med 2024;137:210–217.
[2] Kaplan DE, Ripoll C, Thiele M, et al. AASLD Practice Guidance on risk stratification and management of portal hypertension and varices in cirrhosis. Hepatology 2024;79:1180–1211.
[3] Sengstaken RW, Blakemore AH. Balloon tamponage for the control of hemorrhage from esophageal varices. Ann Surg 1950;131:781–789.
[4] Hunt PS, Korman MG, Hansky J, et al. An 8-year prospective experience with balloon tamponade in emergency control of bleeding esophageal varices. Dig Dis Sci 1982;27:413–416.
[5] Keung CY, Morgan A, Le ST, et al. Survival outcomes and predictors of mortality, re-bleeding and complications for acute severe variceal bleeding requiring balloon tamponade. World J Hepatol 2022;14:1584–1597.
[6] Zehetner J, Shamiyeh A, Wayand W, et al. Results of a new method to stop acute bleeding from esophageal varices: implantation of a self-expanding stent. Surg Endosc 2008;22:2149–2152.
[7] Pfisterer N, Riedl F, Pachofszky T, et al. Outcomes after placement of a SX-ELLA oesophageal stent for refractory variceal bleeding-a national multicentre study. Liver Int 2019;39:290–298.
[8] Lo GH. The use of esophageal stent in controlling acute refractory variceal bleeding. Hepatology 2017;65:385–386.
[9] Songtanin B, Kahathuduwa C, Nugent K. Esophageal stent in acute refractory variceal bleeding: a systematic review and a meta-analysis. J Clin Med 2024;13:357.
[10] de Franchis R, Bosch J, Garcia-Tsao G, et al. Baveno VII - renewing consensus in portal hypertension. J Hepatol 2022;76:959–974.

[11] Escorsell A, Pavel O, Cardenas A, et al. Esophageal balloon tamponade *vs.* esophageal stent in controlling acute refractory variceal bleeding: a multicenter randomized, controlled trial. Hepatology 2016;63:1957–1967.

[12] Rudler M. Management of refractory variceal bleeding. Cham: Springer International Publishing; 2022. p. 477–483.

[13] Garcia-Pagan JC, Caca K, Bureau C, et al. Early use of TIPS in patients with cirrhosis and variceal bleeding. N Engl J Med 2010;362:2370–2379.

[14] Rodrigues SG, Cardenas A, Escorsell A, et al. Balloon tamponade and esophageal stenting for esophageal variceal bleeding in cirrhosis: a systematic review and meta-analysis. Semin Liver Dis 2019;39:178–194.

[15] Kumar R, Kerbert AJC, Sheikh MF, et al. Determinants of mortality in patients with cirrhosis and uncontrolled variceal bleeding. J Hepatol 2021;74:66–79.

[16] de Franchis R, Baveno VIF. Expanding consensus in portal hypertension: report of the Baveno VI Consensus Workshop: stratifying risk and individualizing care for portal hypertension. J Hepatol 2015;63:743–752.

[17] Thabut D, Pauwels A, Carbonell N, et al. Cirrhotic patients with portal hypertension-related bleeding and an indication for early-TIPS: a large multicentre audit with real-life results. J Hepatol 2017;68:73–81.

[18] Haute Autorité de Santé. Removable covered self-expanding esophageal stent (SX-ELLA Stent Danis) and its extraction system (ELLA Extractor). www.has-sante.fr/jcms/p_3211567/fr/danis-stent. [Accessed 4 September 2025].

[19] Weil D, Thabut D, Hernandez-Gea V, et al. Rescue TIPS (rTIPS) must be considered as soon as a tamponade is used: results from two international multicenter cohorts of 3019 patients with portal hypertension (PHT)-related bleeding. Hepatology 2022;76:S151–S154.

Keywords: Portal hypertension; Esophageal varices; Tamponade devices; Rescue transjugular intrahepatic portosystemic shunt (rTIPS); Mortality.

Received 17 January 2025; received in revised form 13 August 2025; accepted 25 August 2025; Available online 30 August 2025

Journal of Hepatology, Volume *7*

Supplemental information

Improved survival in patients with refractory variceal bleeding treated with esophageal stents: A multicenter cohort study

Delphine Weil, Morgane Clément, Charlotte Bouzbib, Jean-Paul Cervoni, Andrimalala Raoto, Grégoire Boivineau, Isabelle Ollivier-Hourmand, Noémi Reboux, Caroline Lemaitre, Cassandra Rayer, Marine Camus-Duboc, Ludovic Caillo, André-Jean Remy, Laure Elkrief, Guillaume Conroy, Faustine Wartel, Armand Garioud, Maeva Guillaume, Edouard Bardou-Jacquet, Stéphane Koch, Jean-Pierre Arpurt, Marika Rudler, Vincent Di Martino, and ANGH, CREGG, SFED, GRAPHE, CFHTP groups

Improved survival in patients with refractory variceal bleeding treated with esophageal stents: A multicenter cohort study

Delphine Weil, Morgane Clément, Charlotte Bouzbib, Jean-Paul Cervoni, Andrimalala Raoto, Grégoire Boivineau, Isabelle Ollivier-Hourmand, Noémi Reboux, Caroline Lemaitre, Cassandra Rayer, Marine Camus-Duboc, Ludovic Caillo, André-Jean Remy, Laure Elkrief, Guillaume Conroy, Faustine Wartel, Armand Garioud, Maeva Guillaume, Edouard Bardou-Jacquet, Stéphane Koch, Jean-Pierre Arpurt, Marika Rudler, Vincent Di Martino, ANGH, CREGG, SFED, GRAPHE, CFHTP groups

Table of contents

Supplementary material 1 2
Table S1 2
Table S2 4
Fig. S1 5
Fig. S2 6
Fig. S3 7
Fig. S4 8
Fig. S5 9
Table S3 10
Table S4 11
Table S5 12
Table S6 13

Supplementary material 1: Construction and Implementation of the Propensity Score Model for Treatment Effect Adjustment

S1A: Development and Selection of the Propensity Score Model

Given the observational design of the study and baseline heterogeneity between treatment groups, we constructed multiple propensity score (PS) models to adjust for confounding in the comparison of outcomes between patients treated with esophageal stents (ES) and those receiving balloon tamponade (BT). Eleven candidate models (PS1 to PS11) were developed using logistic regression, each incorporating different combinations of up to 36 baseline covariates.

To ensure robustness, only variables available prior to treatment initiation were included, while those showing multicollinearity or clinical redundancy were excluded. Model performance was assessed based on two key metrics of covariate balance after weighting: (i) the mean standardized mean difference (SMD) across all covariates, and (ii) the number of covariates achieving an SMD below the accepted threshold of 0.2.

Inverse probability of treatment weighting (IPTW) was computed as 1/PS for treated patients and 1/(1–PS) for controls. Stabilized weights were calculated using the marginal probability of treatment, and all weights were truncated at a maximum of 10 to minimize variance inflation. Among all models tested, PS3 demonstrated the best overall balance, with a mean SMD of 0.453 and 23 out of 36 covariates adequately balanced after applying stabilized and truncated IPTW (stIPTW). These results are summarized in the table below.

Table S1

PS Model	Number of included variables	AUROC	95% CI AUROC	Mean SMD (crude IPTW)	Number of Variables with SMD < 0.2 (crude IPTW)	Mean SMD (stabilized & truncated IPTW)	Number of variables with SMD < 0.2 (stabilized & truncated IPTW)
**PS1**	18	0.873	[0.766 – 0.953]	0.634	12	0.610	14
**PS2**	22	0.909	[0.832 – 0.969]	2.442	2	0.476	21
**PS3**	23	0.935	[0.865 – 0.979]	2.212	1	**0.453**	**23**
**PS4**	24	0.944	[0.885 – 0.983]	1.994	1	0.455	23
**PS5**	23	0.923	[0.851 – 0.978]	2.315	2	0.468	22
**PS6**	22	0.907	[0.827 – 0.969]	2.665	1	0.497	20
**PS7**	23	0.938	[0.868 – 0.982]	2.064	3	0.458	22
**PS8**	21	0.888	[0.795 – 0.960]	3.07	1	0.510	20
**PS9**	20	0.841	[0.742 – 0.927]	3.027	2	0.514	24
**PS10**	21	0.846	[0.738 – 0.932]	2.868	2	0.517	23
**PS11**	22	0.877	[0.779 – 0.955]	2.635	4	0.501	23

S1B: Specification and Predictive Performance of PS3

The propensity score for receiving esophageal stenting was estimated using a multivariable logistic regression model (PS3) including 23 covariates. The logit of the PS3 was calculated as:

Logit(PS3) *= 0.1809 ×* ***Age*** *− 11.3146 × (****General anesthesia*** *= 1) + 0.2967 ×* ***Albumin*** *− 0.0157 ×* ***Year of inclusion*** *− 1.4443 × (****Ascites*** *= 1) − 1.5522 × (****History of portal hypertension-related bleeding*** *= 1) + 1.7964 × (****Known esophageal varices*** *= 1) − 4.8057 × (****Metabolic cirrhosis*** *= 1) − 8.3885 × (****University hospital*** *= 1) + 0.0212 ×* ***Creatinine*** *+ 1.0677 ×* ***Time from bleeding onset to tamponade*** *− 1.5524 × (****Hepatic encephalopathy*** *= 1) − 0.6684 ×* ***Hemoglobin*** *+ 11.5724 × (****Intubation*** *= 1) − 2.1158 × (****Vasopressors*** *= 1) − 0.1420 ×* ***MELD score*** *+ 0.1602 ×* ***RBC units transfused on day 0*** *+ 0.0788 ×* ***Mean arterial pressure*** *+ 2.8440 × (****ICU admission*** *= 1) + 3.7778 × (****Prospective enrollment*** *= 1) + 2.1347 ×* ***Child-Pugh score*** *− 0.2768 × (****Male sex*** *= 1) − 1.4305 × (****Beta-blocker treatment*** *= 1).*

The individual probability (PS) was then obtained by applying the standard logistic transformation: PS = exp(Logit) / [1 + exp(Logit)].

The figure below illustrates the receiver operating characteristic (ROC) curve showing the discriminative ability of the PS3 model to predict the use of esophageal stenting. The model demonstrated excellent performance, with an area under the ROC curve (AUROC) of 0.935 (95% CI: 0.876–0.981), confirming its strong ability to distinguish between patients who received esophageal stenting and those treated with balloon tamponade.

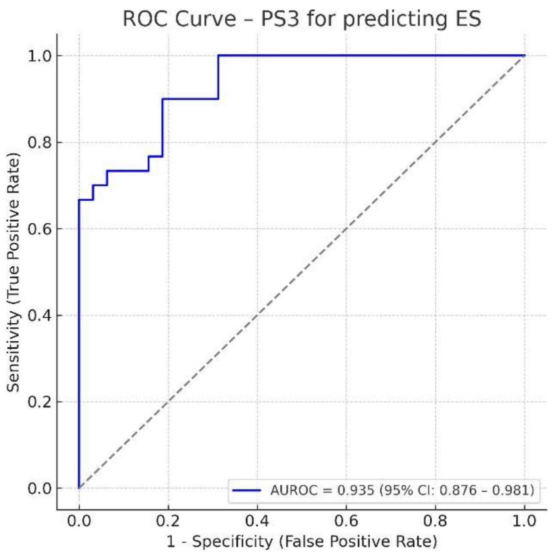

S1C: Weighting and Adjustment Strategy

To evaluate the effectiveness of the weighting strategy derived from the PS3 model, we assessed the standardized mean differences (SMDs) for key baseline covariates before and after weighting. The table below presents SMDs in the unweighted sample, after crude IPTW (IPTW3), and after application of stabilized and truncated IPTW (stIPTW3), with weights capped at 10. While crude IPTW sometimes exacerbated imbalance—with several covariates exceeding the conventional SMD threshold of 0.5—stabilized and truncated IPTW3 consistently improved covariate balance across all variables. Most SMDs fell below 0.2, indicating satisfactory adjustment and supporting the use of stIPTW3 for all subsequent outcome analyses.

Table S2.

Variable	SMD unweighted	SMD with crude IPTW3	SMD with stabilized and truncated PTW3
Hepatic encephalopathy	0,008	1,323	0,003
Melena	0,125	1,000	0,032
Alcoholic cirrhosis	0,021	0,440	0,037
Nb of RBC packs transfused	0,041	2,482	0,058
MASLD-related cirrhosis	0,039	0,796	0,058
History of Hepatic encephalopathy	0,203	0,190	0,068
Ascites	0,144	1,051	0,071
APAs	0,017	0,693	0,073
Pugh score	0,375	1,697	0,079
Hematemesis	0,112	1,083	0,079
Age	0,014	0,510	0,088
Male	0,079	0,459	0,090
Transfusion	0,021	0,695	0,104
Known EVs	0,214	0,572	0,109
Prospective enrollment	0,517	1,390	0,110
Vasopressors	0,000	1,073	0,116
ICU admission	0,134	1,031	0,116
Pharmacologic Treatment of variceal bleeding	0,100	0,701	0,125
Intubation	0,357	0,749	0,140
ß-Blockers	0,256	1,159	0,142
Year of enrollment	0,596	0,856	0,151
Albumin	0,089	2,526	0,165
TIPS prior index bleeding	0,372	1,734	0,174
History of PTH-related GI bleeding	0,129	0,464	0,181
General Anesthesia	0,428	1,230	0,218
MAP	0,220	1,470	0,221
Prothrombin time	0,314	1,285	0,242
Hemoglobin	0,507	0,300	0,263
Bilirubin	0,202	0,748	0,264
Diuretics	0,256	1,484	0,289
Rectorrhagia	0,164	0,308	0,345
Creatinine	0,181	0,565	0,406
Endoscopy on day 0	0,582	0,281	0,475
MELD score	0,361	1,651	0,480
University Hospital	0,771	0,776	0,564
History of ascites	0,448	1,438	0,604

Abbreviations: APAs: Anti-platelet agents; EVs: esophageal varices; GI: gastro-intestinal; ICU: intensive care unit; MAP: mean arterial pressure; MASLD: Metabolic dysfunction–Associated Steatotic Liver Disease; MELD: model for end-stage liver disease; PHT: portal hypertension; TIPS: transjugular intrahepatic portosystemic shunt; RBC: red blood cell.

Fig. S1: Patient inclusion timeline by center and treatment

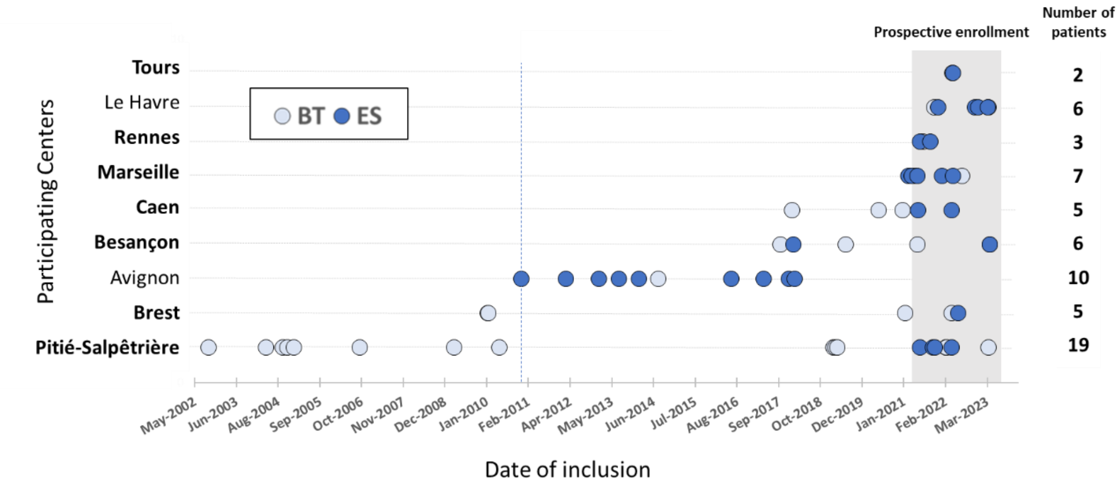

Each dot represents a patient included in the study, positioned according to the date of inclusion (X-axis) and the participating center (Y-axis). All centers used both Balloon Tamponade (BT, light blue dots) and Esophageal Stent (ES, dark blue dots). The vertical dashed line indicates the beginning of the period during which ES was available. Centers affiliated with university hospitals are shown in bold. The grey-shaded area indicates the period during which inclusions were conducted prospectively. The total number of patients included in each center is shown in the right-hand column.

Fig. S2: Week-6 survival in the ES and BT groups. Sensitivity analysis restricted to the period from December 2010 to April 2023.

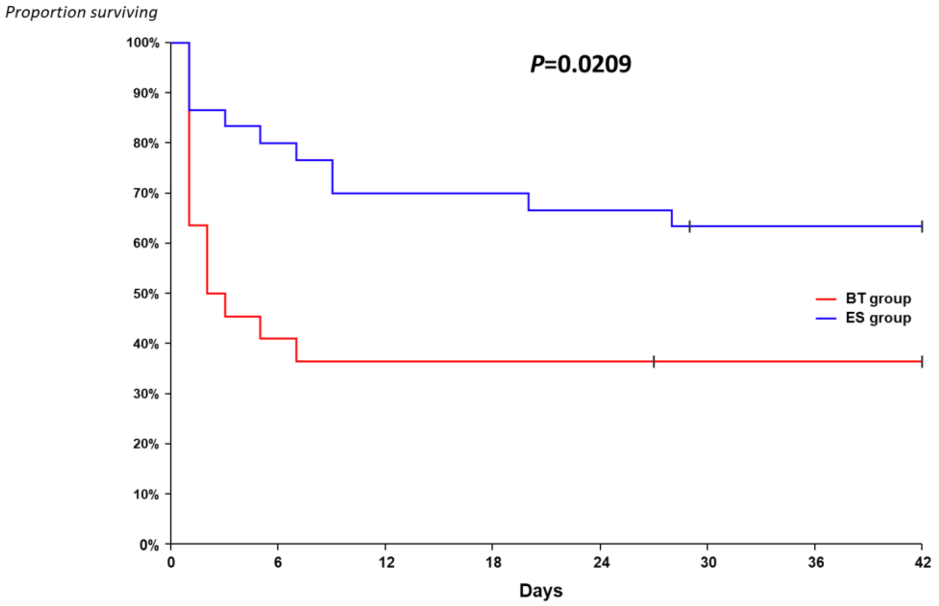

Sensitivity analysis restricted to the period from December 2010 to April 2023, during which esophageal stenting was progressively introduced across centers. Only the 53 patients included during periods and in centers where ES was available were analyzed. Six-week survival remained significantly higher in the ES group (Log-rank test, p= 0.0209), supporting the robustness of the association.

Fig. S3: Impact of rescue transjugular intrahepatic portosystemic shunt (rTIPS) on week-6 survival

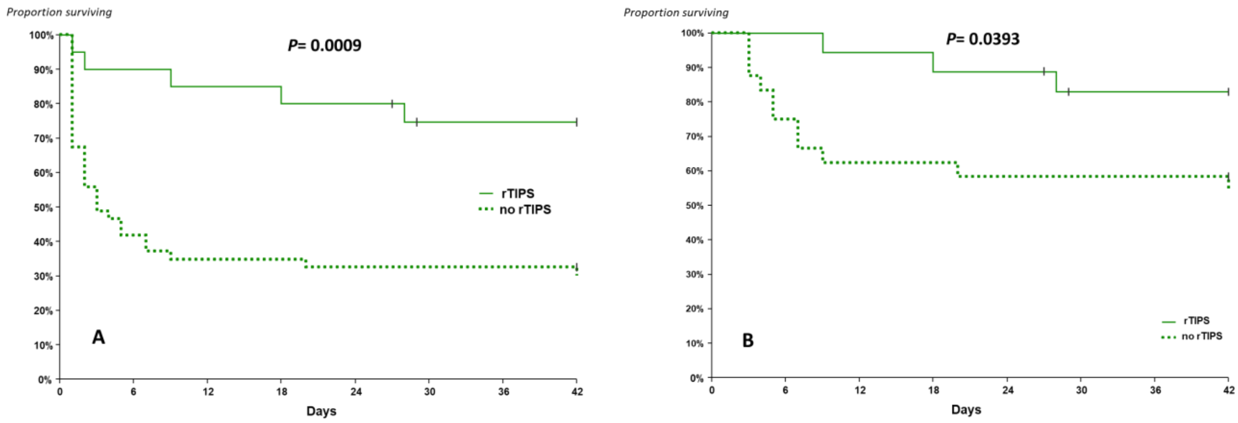

These figures illustrate the protective impact of rTIPS on week-6 mortality.

A: Analysis of the entire study population.

B: Landmark sensitivity analysis to minimize immortal time bias related to TIPS. Because 90% of rescue TIPS were performed within the first two days, this analysis excluded all patients who died before day 2. The solid green line indicates patients who underwent rTIPS, whereas the dotted green line indicates patients who did not undergo rTIPS. The p-values are derived from log-rank tests.

Fig. S4: Comparison of week-6 mortality between ES and BT groups among subgroups

Subgroup	ES	BT	Odds Ratio [95% CI]
	no. deaths at wk 6 / total no.		
Age			
24 to 59	8/22	16/22	0.21 [0.06-0.77]
60 to 86	3/8	8/11	0.23 [0.03-1.58]
Sex			
male	10/27	21/28	0.20 [0.06-0.62]
female	1/3	3/5	0.33 [0.02-6.65]
Prospective enrollment			
Yes	8/19	7/12	0.52 [0.12-2.25]
No	3/11	17/21	0.09 [0.02-0.49]
Alcohol-related cirrhosis			
Yes	8/27	24/30	0.11 [0.03-0.36]
No	3/3	0/3	NC
Past History of variceal bleeding			
Yes	5/16	9/15	0.30 [0.07-1.33]
No	6/14	15/18	0.15 [0.03-0.77]
Child-Pugh Stage			
A/B	3/10	5/7	0.17 [0.02-1.44]
C	8/20	18/25	0.26 [0.07-0.90]
MELD score			
6 to 19.9	3/15	7/14	0.25 [0.05-1.29]
20 to 40	8/15	14/16	0.16 [0.03-0.98]
Creatinine (µmol/L)			
35 to 99	5/17	9/16	0.32 [0.08-1.36]
100 to 581	6/13	13/15	0.13 [0.02-0.83]
Hematemesis			
Yes	7/23	19/27	0.18 [0.05-0.62]
No	4/7	5/6	0.27 [0.02-3.65]
Hospital			
General hospital	2/13	1/3	0.36 [0.02-6.19]
University hospital	9/17	23/30	0.34 [0.10-1.22]
Admission to ICU			
Yes	7/17	10/16	0.42 [0.10-1.70]
No	4/13	14/17	0.10 [0.02-0.53]
Endoscopic treatment prior to tamponade			
Yes	8/18	13/19	0.37 [0.10-1.41]
No	3/11	6/8	0.12 [0.02-1.00]
Rescue TIPS after tamponade			
Yes	3/12	2/8	1.00 [0.13-7.89]
No	8/18	22/25	0.11 [0.02-0.50]

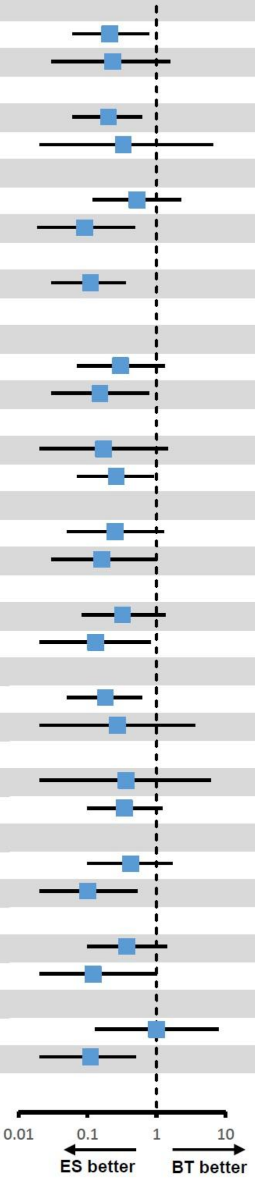

Subgroup analyses of week-6 mortality comparing esophageal stenting (ES) and balloon tamponade (BT). Odds ratios favoring ES were consistently observed across most subgroups, despite small sample sizes. The protective effect of ES was particularly marked in patients aged <60, with alcohol-related cirrhosis, high MELD or creatinine levels, and in those not admitted to the intensive care unit (ICU). In patients not receiving rescue transjugular intrahepatic portosystemic shunt (TIPS), ES was significantly associated with lower mortality, whereas no benefit was seen in those who subsequently received TIPS, suggesting that the survival advantage of ES is mainly observed when TIPS is not performed. Some confidence intervals are wide, reflecting limited power, but overall results support the robustness of ES efficacy across clinically relevant subgroups. The univariate analyses were performed with Chi-square tests.

Fig. S5: Respective impact of esophageal stenting (vs. balloon tamponade) and rescue TIPS on week-6 mortality

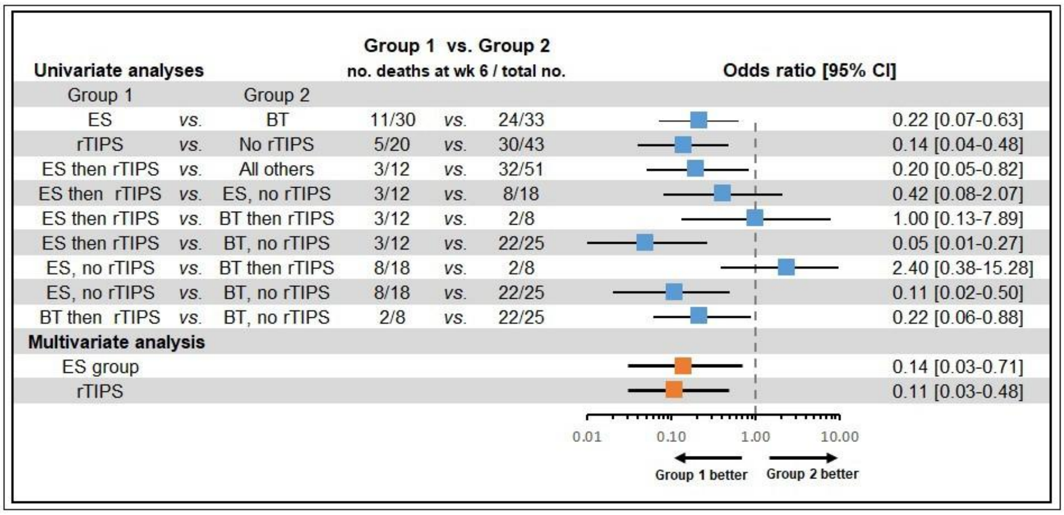

Multivariable logistic regression adjusted for MELD score and stabilized and truncated inverse probability of treatment weighting (stIPTW) (orange markers, lines 10 and 11) confirmed that both esophageal stenting (ES) and rescue TIPS (rTIPS) independently reduce six-week mortality. Despite limited subgroup sizes, two consistent findings emerged: ES was significantly more protective than BT in the absence of rTIPS (line 8), and rTIPS was associated with improved survival across all subgroups, including those initially managed with BT (lines 2, 4, 6, 7, and 9). Once rTIPS was placed, the additional benefit of prior ES was no longer apparent (line 5). The absence of a significant difference between patients treated with rTIPS and those who received ES without rTIPS (lines 4 and 7) likely reflects a type II error, and should not lead us to mistakenly conclude that early esophageal stenting obviates the need for timely rescue TIPS. The univariable analyses used Chi-squared tests.

Table S3: Multivariable time-dependent Cox regression analysis of Factors Associated with week-6 mortality

Model 63 patients; 35 events. r^2 (model) = 0.339.

Variables	HR	95%CI HR	r^2	p
**Esophageal Stent**	0.261	0.097 – 0.702	0.333	0.0078
**Rescue TIPS**	0.211	0.059 – 0.752	0.350	0.0165
**MELD score***	1.056	1.018 – 1.094	0.313	0.0035
**stIPTW3***	0.978	0.880 – 1.086	0.011	0.6719

TIPS : transjugular portosystemic shunt; MELD: model for end stage liver disease; HR: hazard ratio; CI: confidence interval; r^2: variance explained. stIPTW: stabilized and truncated Inverse probability of Treatment (Esophageal Stent) Weighting (*expressed as continuous variables).

TIPS was modeled as a time-dependent covariate to mitigate immortal time bias. Esophageal stent use and TIPS were both independently associated with reduced 6-week mortality, as well as low MELD scores. Model performance: concordance = 0.757; pseudo-R^2 = 0.339.

Mutivariable time-dependent Cox regression model weighted by stIPTW3 (instead of incorporating stIPTW3 as covariate) showed similar results for Esophageal Stent (HR = 0.269; 95%CI: 0.119-0.605; p = 0.0015) and MELD score (HR = 1.060; 95%CI: 1.011-1.110; p = 0.0129), but the effect of rescue TIPS was no longer significant (HR = 0.274; 95%CI: 0.056-1.349;p = 0.1120).

Table S4: Multivariable Logistic Regression Analyses of Factors Associated with Successful Bleeding Control at Day 5.

R^2 (model) = 0.333

Variables	OR	95%CI OR	p
**Esophageal Stent**	6.127	1.323– 28.376	0.0205
**Rescue TIPS**	3.955	1.065 – 14.689	0.0145
**MELD score**	0.919	0.871 – 0.970	0.0400
**stIPTW3**	1.076	0.898 – 1.288	0.4270

Mutivariable Logistic regression model weighted by stIPTW3 (instead of incorporating stIPTW3 as covariate) showed similar results for Esophageal Stent (HR=14.716; 95%CI: 3.145-68.865; p=0.0006) and MELD score (HR=0.920; 95%CI: 0.871-0.971; p=0.0027), but the effect of TIPS was no longer significant (HR=2.553; 95%CI: 0.468-13.910; p=0.2786).

Table S5: Sensitivity analyses on week-6 mortality: assessing the robustness of the esophageal stenting effect and correcting for TIPS-related immortal time bias

S5A: Time-dependent Cox multivariable model of week-6 mortality in subjects registered from December 2010 to April 2023 (time frame during which the esophageal stent was available).

Variables	HR	95%CI HR	p
**Esophageal Stent**	0.238	0.081 – 0.694	0.0086
**Rescue TIPS**	0.166	0.035 – 0.794	0.0217
**MELD score***	0.919	0.871 – 0.970	0.0400
**stIPTW3**	0.997	0.877 – 1.133	0.9583

52 patients ; 25 events ; R^2(model) = 0.235

TIPS was modeled as a time-dependent variable using a start–stop Cox model to accurately account for the time at risk before TIPS placement and prevent overestimation of its protective effect. The analysis covers patients who were enrolled during the period when the esophageal stent was available, either after its commercial release or before, at pilot centers.

S5B: Cox model for week-6 mortality restricted to the 42 patients who survived two days

Variables	HR	95%CI HR	p
**Esophageal Stent**	0.206	0.059 – 0.720	0.0134
**Rescue TIPS**	0.235	0.061 – 0.901	0.0347
**MELD score***	1.096	1.032 – 1.163	0.0028

42 patients; 14 events; R^2(Model) = 0.306

To account for potential residual immortal time bias—arising from early deaths that inherently preclude TIPS placement—we performed a landmark analysis restricted to patients who were still alive on day 2. This time point was selected because 90% of rescue TIPS procedures were performed within the first 48 hours after the bleeding episode. Restricting the cohort in this way ensures that all included patients had a real and comparable opportunity to receive the intervention, thus enhancing the validity of comparisons between the TIPS and no-TIPS groups. Given the limited number of events, only the model without adjustment for stIPTW3 is reported here. When this fourth covariate was included, the model converged but attenuated the statistical significance of the ES variable (p = 0.0501), despite a similar effect size (HR = 0.228).

Table S6: Multivariable sensitivity analyses for robustness assessment of the effect of esophageal stenting on early bleeding control and week-6 mortality: modifications of the adjustment covariate.

Logistic regression models for Control of initial bleeding	OR	95%CI OR	p
unadjusted model			
ES	8.999	2.626-30.872	0.0005
TIPS	4.016	1.085-14.863	0.0373
MELD score (continuous)	0.931	0.892-0.971	0.0009
model adjusted on propensity score			
ES	6.412	1.548-26.561	0.0104
TIPS	3.381	0.872-13.107	0.0781
MELD score (continuous)	0.913	0.860-0.970	0.0033
PS3	2.433	0.363-16.326	0.3599
model adjusted on crude IPTW			
ES	9.736	2.541-37.300	0.0009
TIPS	3.737	0.998-13.986	0.0503
MELD score (continuous)	0.932	0.894-0.972	0.0011
cIPTW3	1.000	0.608-1.645	1.0000
model adjusted on stabilized and truncated IPTW			
ES	6.127	1.323-28.376	0.0205
TIPS	3.955	1.065-14.689	0.04
MELD score (continuous)	0.919	0.871-0.970	0.0023
stIPTW3	1.076	0.898-1.288	0.4271

Cox models for 6-week mortality	HR	95%CI HR	p	r^2
Unadjusted model				
ES	0.254	0.117-0.551	0.0005	0.275
TIPS	0.195	0.067-0.565	0.0026	0.180
MELD score (continuous)	1.049	1.012-1.088	0.0083	0.222
Model adjusted on the propensity score				
ES	0.291	0.116-0.728	0.0083	0.185
TIPS	0.207	0.070-0.616	0.0046	0.207
MELD score (continuous)	1.052	1.013-1.092	0.0078	0.187
PS3	0.716	0.231-2.217	0.5624	0.011
Model adjusted on the crude IPTW				
ES	0.239	0.107-0.536	0.0005	0.283
TIPS	0.197	0.068-0.574	0.0029	0.224
MELD score (continuous)	1.049	1.012-1.088	0.0093	0.181
cIPTW3	1,000	1.000-1.000	0.4774	0.016
Model adjusted on the stabilized and truncated IPTW				
ES	0.276	0.102-0.748	0.0114	0.173
TIPS	0.195	0.067-0.567	0.0027	0.227
MELD score (continuous)	1.049	1.012-1.087	0.0094	0.18
stIPTW3	0.986	0.898-1.095	0.7954	0.002

To assess the robustness of our findings, we constructed multivariable models for the two primary endpoints—bleeding control on day 5 and week-6 mortality—using four adjustment strategies: unadjusted; adjusted for the propensity score (PS3); weighted using crude IPTW (cIPTW3); and weighted using stabilized and truncated IPTW (stIPTW3). The models included the following covariates: tamponade modality (esophageal stent [ES] vs. balloon tamponade [BT]), rescue TIPS, MELD score, and the adjustment variable (PS or IPTW).

The benefit of ES for bleeding control was strong and consistent. The unadjusted odds ratio (OR) was 8.999 (95% CI: 2.626–30.872, p = 0.0005), and remained significant after full adjustment with stIPTW3 (OR = 5.981, 95% CI: 1.624–22.024, p = 0.0073), indicating a robust independent effect of ES on early bleeding control. MELD score was inversely associated with bleeding control across all models. The effect of rescue TIPS was more variable: significant in the unadjusted and stIPTW3 models, but not in models adjusted for PS3 or using cIPTW3. This suggests that poorly specified adjustments may obscure true effects, either by increasing imbalance (as seen with cIPTW) or failing to address residual confounding (as with PS adjustment). In contrast, stIPTW3 restored both covariate balance and interpretability, revealing the protective effect of rescue TIPS otherwise masked by indication bias.

For week-6 mortality, results were more consistent. ES remained protective across all models (unadjusted HR=0.254, 95% CI: 0.117–0.551, p = 0.0005; stIPTW3-adjusted HR=0.276, 95% CI: 0.102–0.748, p = 0.0114). Rescue TIPS was also independently associated with improved survival in all configurations, including the fully adjusted model (HR = 0.195, 95% CI: 0.067–0.567, p = 0.0027). MELD remained a consistent predictor of mortality.

Importantly, all Cox models were non–time-dependent and did not account for the immortal time bias introduced by delayed TIPS. Since 90% of procedures occurred within 48 hours, most patients who died before day 2 could not receive TIPS. By not correcting for this bias, the analyses intentionally overestimated the apparent benefit of TIPS, thereby placing the initial tamponade modality (i.e., ES or BT) at a comparative disadvantage. The persistence of a significant association between esophageal stenting and improved week-6 survival under these analytically conservative conditions further reinforces the robustness of the observed benefit of esophageal stents.
